# A geospatiotemporal and causal inference epidemiological exploration of substance and cannabinoid exposure as drivers of rising US pediatric cancer rates

**DOI:** 10.1186/s12885-021-07924-3

**Published:** 2021-02-25

**Authors:** Albert Stuart Reece, Gary Kenneth Hulse

**Affiliations:** 1 Division of Psychiatry, University of Western Australia, Crawley, Western Australia 6009 Australia; 2 School of Medical and Health Sciences, Edith Cowan University, Joondalup, Western Australia 6027 Australia

**Keywords:** Cannabis, Cannabinoid, Δ9-tetrahydrocannabinol, Cannabigerol, Genotoxicity, Acute leukaemia, Pediatric cancer

## Abstract

**Background:**

Age-adjusted US total pediatric cancer incidence rates (TPCIR) rose 49% 1975–2015 for unknown reasons. Prenatal cannabis exposure has been linked with several pediatric cancers which together comprise the majority of pediatric cancer types. We investigated whether cannabis use was related spatiotemporally and causally to TPCIR.

**Methods:**

State-based age-adjusted TPCIR data was taken from the CDC Surveillance, Epidemiology and End Results cancer database 2003–2017. Drug exposure was taken from the nationally-representative National Survey of Drug Use and Health, response rate 74.1%. Drugs included were: tobacco, alcohol, cannabis, opioid analgesics and cocaine. This was supplemented by cannabinoid concentration data from the Drug Enforcement Agency and ethnicity and median household income data from US Census.

**Results:**

TPCIR rose while all drug use nationally fell, except for cannabis which rose. TPCIR in the highest cannabis use quintile was greater than in the lowest (β-estimate = 1.31 (95%C.I. 0.82, 1.80), *P* = 1.80 × 10^− 7^) and the time:highest two quintiles interaction was significant (β-estimate = 0.1395 (0.82, 1.80), *P* = 1.00 × 10^− 14^). In robust inverse probability weighted additive regression models cannabis was independently associated with TPCIR (β-estimate = 9.55 (3.95, 15.15), *P* = 0.0016). In interactive geospatiotemporal models including all drug, ethnic and income variables cannabis use was independently significant (β-estimate = 45.67 (18.77, 72.56), *P* = 0.0009). In geospatial models temporally lagged to 1,2,4 and 6 years interactive terms including cannabis were significant. Cannabis interactive terms at one and two degrees of spatial lagging were significant (from β-estimate = 3954.04 (1565.01, 6343.09), *P* = 0.0012). The interaction between the cannabinoids THC and cannabigerol was significant at zero, 2 and 6 years lag (from β-estimate = 46.22 (30.06, 62.38), *P* = 2.10 × 10^− 8^). Cannabis legalization was associated with higher TPCIR (β-estimate = 1.51 (0.68, 2.35), *P* = 0.0004) and cannabis-liberal regimes were associated with higher time:TPCIR interaction (β-estimate = 1.87 × 10^− 4^, (2.9 × 10^− 5^, 2.45 × 10^− 4^), *P* = 0.0208). 33/56 minimum e-Values were > 5 and 6 were infinite.

**Conclusion:**

Data confirm a close relationship across space and lagged time between cannabis and TPCIR which was robust to adjustment, supported by inverse probability weighting procedures and accompanied by high e-Values making confounding unlikely and establishing the causal relationship. Cannabis-liberal jurisdictions were associated with higher rates of TPCIR and a faster rate of TPCIR increase. Data inform the broader general consideration of cannabinoid-induced genotoxicity.

**Supplementary Information:**

The online version contains supplementary material available at 10.1186/s12885-021-07924-3.

## Background

CDC Surveillance, Epidemiology and End Results (SEER) data from 9 US cancer registries indicates that the age-adjusted total Pediatric (age less than 20 years) cancer incidence rate (TPCIR) has risen 49.0% from 12.96 to 19.32 / 100,000 from 1975 to 2015 [1]. Cancer incidence is U-shaped across the pediatric age range being higher in the under 5 years and over 14 years age groups [2]. Leukaemias, brain and nervous system, neuroblastoma, soft tissue sarcoma, lymphoma and testicular cancer are amongst the commonest pediatric cancers [2, 3].

Notwithstanding a generally falling mortality rate from childhood cancer, the TPCIR incidence is acknowledged to be rising since the records of collated cancer registries were first published in 1975 [2]. The cause of this unprecedented increase is at present unclear. Moreover major ethnic differentials are evident for tumours such as All Childhood Cancer (ACC), acute lymphatic leukaemia (ALL) and brain and testicular cancers where the rates in African-American patients vary from 20 to 70% of those in the Caucasian-American community [2]. Again the reasons for such large ethnic disparities are unknown. It therefore appears that several of the major questions relating to the aetiopathogenesis of pediatric cancer are outstanding.

Whilst in adult populations the relationship between cannabis use and cancer incidence is controversial with both positive and negative reports in existence [4, 5], amongst pediatric populations the situation is much clearer. It was noted by the California Environmental Protection Agency in a very detailed literature review that five of six studies reported a positive relationship [6–11]. Parental cannabis use has been linked with acute lymphatic leukaemia, acute myeloid leukaemia, childhood astrocytoma, rhabdomyosarcoma and neuroblastoma [2, 7–12]. Together these comprise 60–70% of the total cancers seen in children younger than 14 years and those between 15 and 20 years [2]. In such a context it becomes plausible that the rise in cannabis use since the 1960’s may be a primary driver of total pediatric cancer.

Testicular cancer is a particularly interesting case. It is well established that testicular cancer occurs mainly in younger men with an age peak at 30–34 years and 20% of cases occur in the pediatric age range [1]. The testes houses the germ cells and cannabinoids are known to have myriad direct effects on the reproductive tract in both sexes [13–17]. There is great uniformity in studies of the cannabis-testicular cancer link as all four studies found a risk elevation of over two-fold [18–21] with an overall risk for current, weekly and chronic smokers of non-seminomatous germ cell tumours estimated in meta-analysis of 2.59 (95%C.I. 1.60–4.19) [22]. Since pediatric cancer often results from inherited genetic errors [23, 24] this implies that major genetic errors in germ cells are induced by parental cannabis exposure.

Adding to concerns related to the potentially genotoxic actions of prenatal cannabinoid exposure (PCE) is an increasing interest in elevation of many birth defects following PCE in Hawaii, Colorado, Canada and Australia [25–28]. A recent report noted a three-fold rise in total congenital defects in the northern Territories of Canada where more cannabis is smoked [28]. Downs syndrome, due to a major genetic trisomic error, has also been found to be elevated following PCE in Hawaii, Colorado and Australia [25–27] and this syndrome has an established link with childhood ALL with 6–10% of Downs syndrome children being affected by this malignancy [29, 30].

As discussed below the physiology and pathophysiology of both the endocannabinoid system and the impacts of diverse exogenous phytocannabinoids is presently being studied in great detail and major impacts on reproductive health, genetic and physiological quality of gametes, epigenetic effects on both DNA methylation and histone synthesis and signalling, immunomodulatory and mitochondriopathic effects, and transgenerational inheritable epigenetic effects in both man and mouse are well established and have been demonstrated by a number of investigators [15, 17, 31–38].

Concerns are heightened by the recent demonstration that 69% of cannabis dispensaries in Colorado recommended cannabis use to pregnant patients for various symptoms in a recent telephone survey [39] and that in 2017 an estimated 161,000 women used cannabis whilst pregnant across USA [40, 41].

Taken together these data suggest that an improved understanding of cannabis-related carcinogenesis in the closely defined pediatric context might well lead to important insights into cannabis-related genotoxicity more generally [42, 43]. Moreover the advent of sophisticated geospatial analysis together with some of the formal techniques of causal inference analysis implies that sophisticated and modern analytical procedures could be brought to bear on these important and increasingly topical issues. Techniques such as inverse probability weighting and e-Values are designed to formally investigate causal, as opposed to merely associational, relationships.

The objective of this study was to determine if the rise in pediatric cancers across USA paralleled the recent rise in the use of cannabis when considered formally across space and time, and if the relationship met the criteria for causal inference when assessed by strict quantitative criteria.

## Methods

### Data

Annual data on age-adjusted rates of pediatric cancer cases occurring in patients less than 20 years old was accessed from the publicly available SEER*Explorer website [1]. Data on state-based pediatric cancer rates was accessed via the SEER*Stat software from the SEER / NCI database [44]. Drug use data was accessed from the nationally representative National Survey of Drug Use and Health (NSDUH) conducted by the Substance Abuse and Mental Health Services Administration (SAMHSA) [45]. This survey reports a 74.1% response rate [46]. Data on the following drug variables was collated: monthly cigarette use; annual alcohol use disorder, monthly cannabis use, annual analgesic abuse and annual cocaine use. Data on ethnic composition and median household income by state and year was accessed via the tidycensus package in R from the US Census Bureau. The ethnicities for which data was collected were: Caucasian American, African American, Hispanic American, Asian American, American Indian / Alaskan Native American, Native Hawaiian / Pacific Islander American. Data on national cannabinoid concentrations for Δ9-tetrahydrocannabinol (THC), cannabinol, cannabigerol and cannabichromene was obtained from various published reports [47–49]. Data on cannabis legal status was adduced from an internet search [50].

### Derived data

Given the clear differences in drug use by ethnicity it was considered important to formally take ethnic cannabis use into account in regression modelling. Data on the frequency of cannabis use by ethnicity was available at the national level from the SAMHSA Substance Abuse and Mental Health Data Archive (SAMHDA) Restricted Use Data Analysis System (RDAS) [45]. For each ethnicity and for each year the percentage of the ethnicity using cannabis at the midpoint of the indicated frequency were multiplied together and summed to gain an ethnic cannabis use index. Hence if fraction x of an ethnicity used cannabis from 20 to 30 days per month then x would be multiplied by 25. This was repeated and summed across all use frequencies to obtain a specific ethnic cannabis use index for that year. This index was multiplied by the state cannabis use rate and the THC concentration in that year to derive an estimate of the ethnic exposure to THC in each state. Similarly the concentration of selected cannabinoids was multiplied by the state cannabis use rate to derive a state based exposure to that cannabinoid. Cannabis use quintiles were defined in each year and concatenated to form strata across all years.

### Missing data

The total pediatric cancer rate for Wyoming 2008 was absent. This was imputed as the mean of its rate in 2007 and 2009. The rate of analgesic use was missing for all states in 2015. This was imputed as the mean of the state rates for 2014 and 2016.

### Statistics

R version 4.0.2 (2020-06-22) from CRAN was used for data analysis and accessed via the RStudio 1.2.5042 (2009–2020) GUI. Data analysis was performed in September 2020. Graphs and map-graphs were drawn using packages ggplot, albersusa and sf. Covariates were log-transformed to approximate normality based on the Shapiro-Wilks test. Linear, mixed effects, panel, robust marginal structural models and spatial models were studied using packages base, nlme, plm, survey and splm (spatial panel linear models) respectively [51–53]. In each case model reduction was performed by the classical technique of serial deletion of the least significant term. A variety of modelling procedures was employed for the following reasons. Mixed effects regression was useful for state-wise study of data, for inverse probability weighted corrections, and for generation of standard deviations which can be input to eValue calculations. Panel regression modelling was well suited to the time series sequential nature of the dataset, can be inverse probability weighted and allowed the use of both lagging and instrumental variables. Robust regression was conducted to examine the robust effects after inverse probability weighting. Spatiotemporal regression was performed as the data are inherently distributed across space and time and there was good evidence from the models for both spatial and temporal autocorrelation (see Results). As the models also produce a variance estimate their output is well suited to the calculation of e-Values. Inverse probability weighting was conducted with the ipw package and e-Values for regression models were calculated with the package EValue. Tests for trend were conducted with the chi squared test in Base. T-tests were conducted for parametric group comparisons and were two tailed. *P* < 0.05 was considered significant throughout.

Panel analysis utilized the pooling technique, a time effect, the random method of Swarmy, the instrumental method of Amemiya and were inverse probability weighted. Robust structural models were conducted by state and were inverse probability weighted.

### Spatial analysis

Interstate geospatial linkages were made on the “queen” basis of shared edges or corners and compiled with the poly2nb function from package spdep. They were edited as described so that no state, such as Alaska or Hawaii, was left geospatially isolated (as shown in Results). Model specification of spatial models was undertaken from the general full model to the specific [54]. That is to say the standard spatiotemporal regression model was conducted using the splm function spreml (spatial panel random effects maximum likelihood) including spatial autocorrelation after Kapoor, Kelejian and Prucha [55], random effects, serial correlation in the residual errors and spatial autocorrelation, coded as sem2srre in spreml models [52]. Significance of the final model parameters phi, psi, rho and lambda which quantify random error, serial correlation in the residuals, spatial error correlation and spatial autocorrelation respectively, confirmed that this maximal structure was appropriate (see Results tables). The spatial error adjustment of Kapoor, Kelejian and Prucha takes into account spatial correlation in both the exposure and the outcome and this was considered to be reflective of the real world situation in this case [54]. spreml models do allow the use of both spatial and temporal lagging which has been utilized as described. At the time of writing splm and spreml spatial models do not allow the use of instrumental variables or inverse probability weighting which implies the need for supplementary techniques.

### Causal inference

Two techniques of causal inference were employed. Inverse probability weights were constructed for the exposure of interest, monthly cannabis exposure, as a function of the other drug variables which were our primary variables of interest. These weights were used to weight mixed effects, panel and robust regression models appropriately. The effect of this procedure is to equalize exposure across study groups and has also been validated for continuous exposures as considered here. Such techniques are said to create pseudo-randomized groups from which causal inferences can properly be made. We also calculated e-Values which are a measure of the association required of any unmeasured potential confounder variable with both the exposure and the outcome to discount the reported results. In the literature minimum (of the two) e-Values above 1.25 are commonly considered of relevance [56].

### Data availability

All data, including R code, inverse probability weights, geospatial weights, and source datasets, has been made publicly available through the Mendeley data base repository and may be accessed at this URL: 10.17632/cnwv9hdspd.1.

### Ethics

The datasets used were all publicly available and de-identified. No reference has been made at any point to individually identifiable data. The present work was approved by the University of Western Australia Human Research Ethics Committee on June 7th 2019 (No. RA/4/20/4724).

## Results

**Fig. 1:**
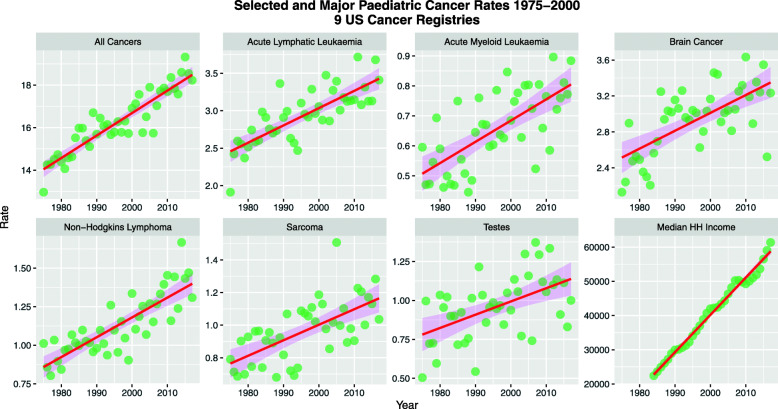
Pediatric Cancers 1975–2017, CDC SEER Explorer Dataset, USA National Level, data derived from 9 cancer registries

Inspection of the SEER*Explorer website shows that at the national level that age-adjusted rates of several cancers in the pediatric age group (younger than 20 years) are rising including all cancer and acute lymphatic leukaemia which is the commonest tumour. The annotation on the SEER website is made from the JoinPoint program which also comes from NCI and CDC. These tumours are listed in Table 1 and illustrated in Fig. 1 using data based on 9 US cancer registries 1975–2017. Supplementary Figure 1 shows other cancers which are mostly rising utilizing data from 21 US cancer registries 2000–2017.

**Table 1:** SEER-Nominated Time Trends of Various Pediatric and Adult Cancers

Cancer	Observed Trend	Delayed Trend
All Pediatric Cancers (< 20 Years)	Rising	Rising
Pediatric ALL - Acute Lymphatic Leukaemia	Rising	
Pediatric AML - Acute Myeloid Leukaemia	Rising	
Pediatric Brain Cancer	Stable	Rising
Pediatric NHL - Non-Hodgkins Lymphoma	Rising	Rising
Sarcoma - All Age	Stable	
Sarcoma < 20 Year - Localized	Rising	
Sarcoma < 20 Year - Distant	Rising	
Sarcoma All Age - Localized	Rising	
Sarcoma All Age - Distant	Rising	
Sarcoma All Age	Rising	
Pediatric Testes < 20 Years	Stable	Stable
Testes < 50 Years	Rising	Rising
Testes All Age	Rising	Rising

**Fig. 2:**
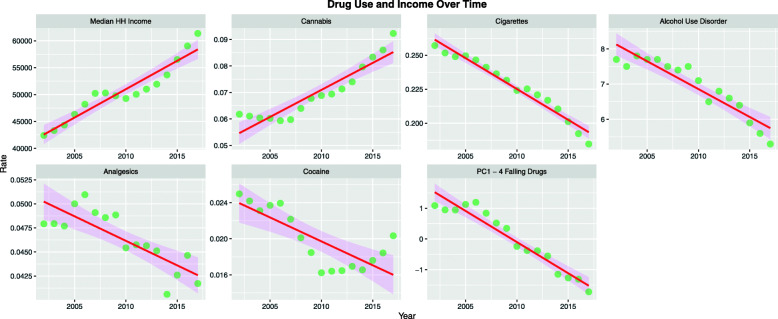
Drug use over time. Data from NSDUH 2002–2017, SAMHSA

Figure 2 shows national drug exposure data from NSDUH 2003–2017 and US Census bureau median household income data. It is important to note that exposure to most classes of drugs is dropping with the notable exception of cannabis. Since SAMHSA NSDUH data could be temporally matched to the CDC SEER cancer database for the years 2003–2017, this became the period of analysis.

**Fig. 3:**
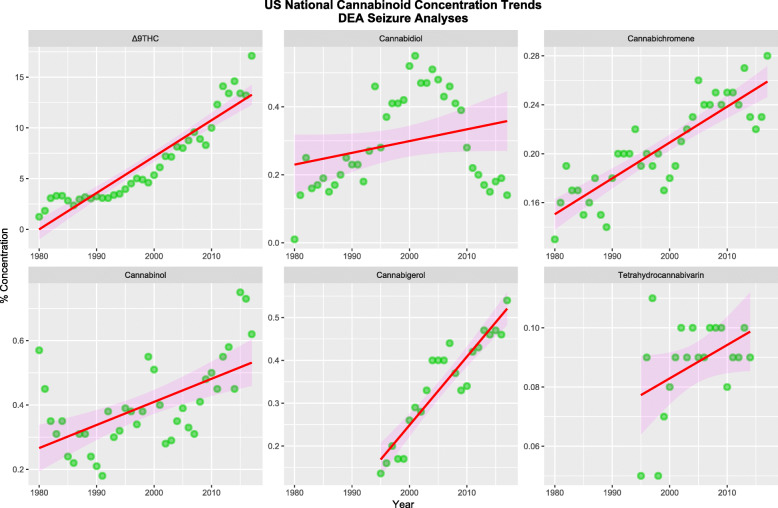
Cannabinoid concentrations in Federal Seizures of Cannabis over time, Drug Enforcement Agency data [47–49]

Figure 3 shows the concentration of various cannabinoids found in federal cannabis seizures 1980–2017 [47–49].

**Fig. 4:**
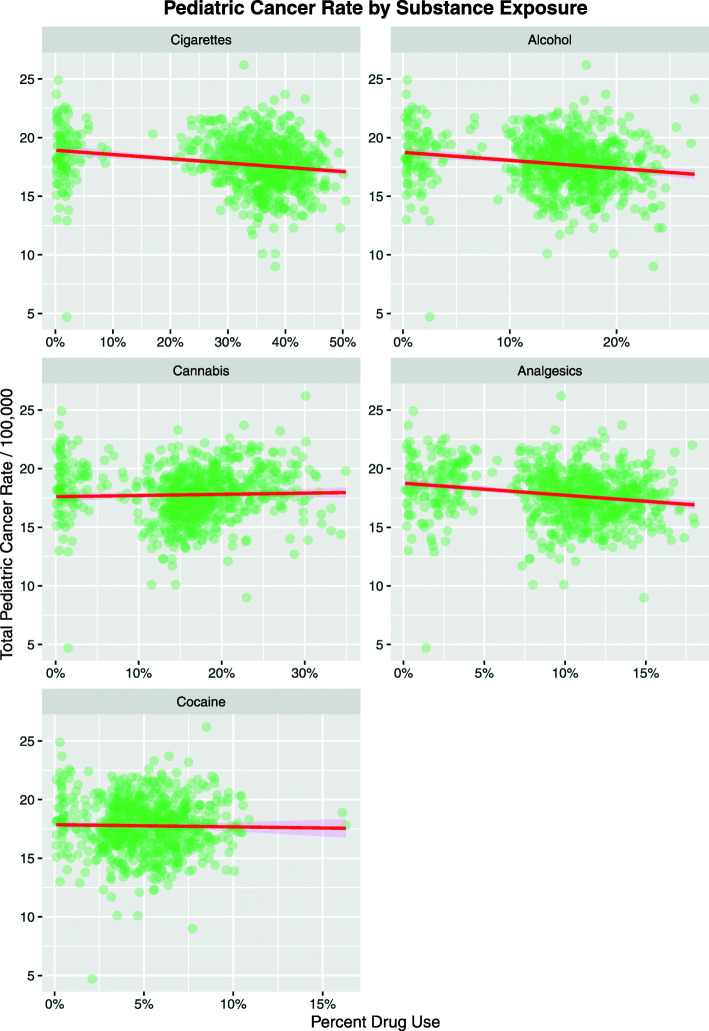
Total pediatric cancer incidence rate as a function of drug exposure

Figure 4 shows the age-adjusted state-based TPCIR plotted as a function of exposure to the various substances listed. The regression line for cannabis is noted to be weakly and non-significantly positive.

**Fig. 5:**
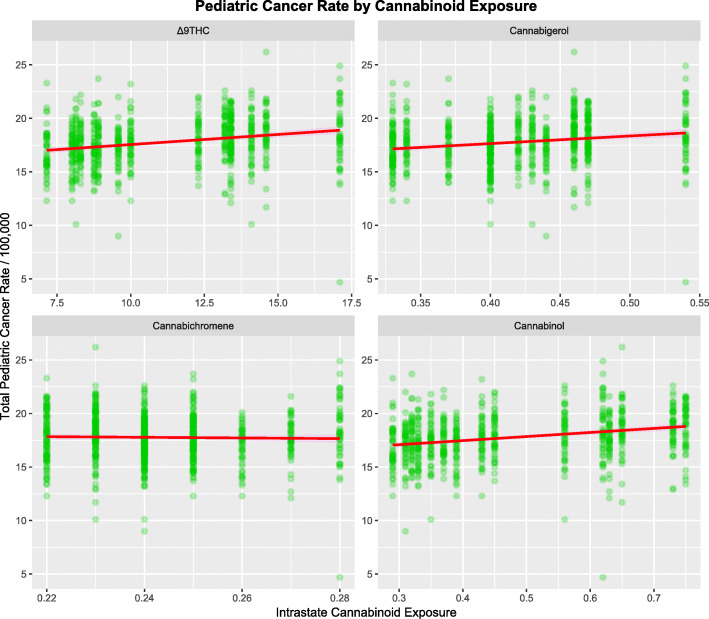
Total pediatric cancer incidence rate as a function of estimated state level cannabinoid exposure

Figure 5 shows plots of the TPCIR rate against selected cannabinoids. The regression lines for THC and cannabigerol appear to be strongly positive.

**Fig. 6:**
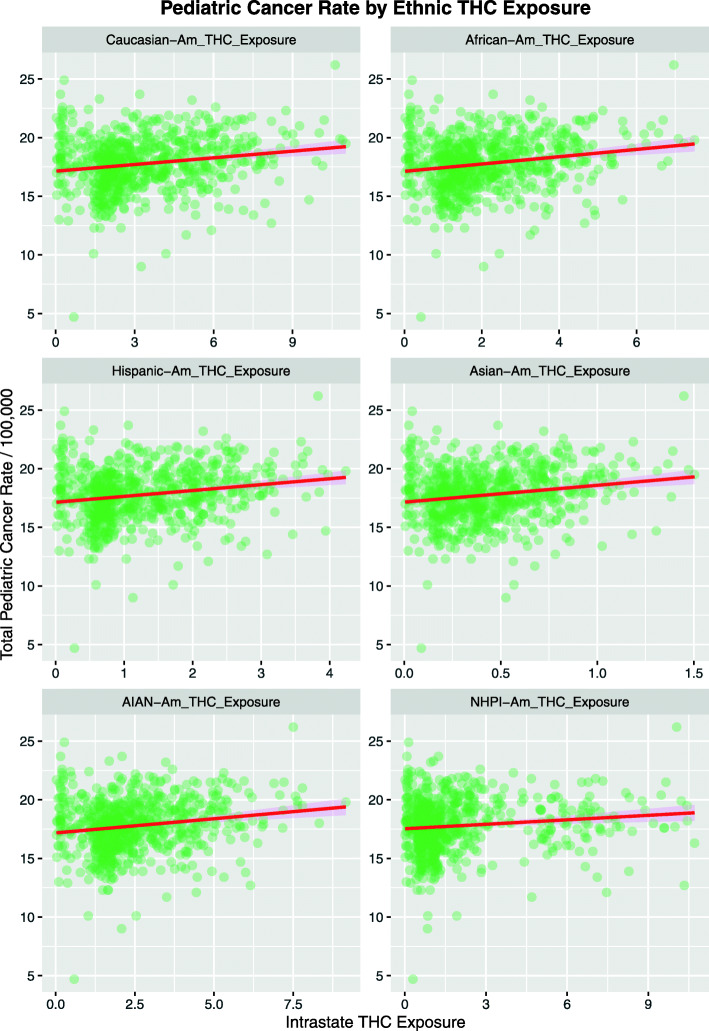
Total pediatric cancer incidence rate as a function of estimated ethnic THC exposure

Figure 6 shows the TPCIR as a function of ethnic cannabis exposure. In each case the regression line appears to be strongly positive and up-sloping.

Table 2 lists applicable results from linear regression against time, cannabis, THC, various substances, cannabinoids and ethnicity. Many results are significant with the notable exception of cannabis.

**Table 2:** Linear Models: TPCIR Against Time, Cannabis, Cannabinoids and Ethnicity

Parameter Estimates			Model Parameters			
Parameter	Estimate (C.I.)	Pr(>|t|)	R-Squared	F	dF	P
***lm(Cancer_Rate ~ Time)***						
Year	0.14 (0.1, 0.17)	3.8E-14	0.0725	59.6	1748	3.80E-14
***lm(Cancer_Rate ~ Cannabis)***						
mrjmon	1.00 (−1.22, 3.22)	0.3800	−0.0003	0.78	1748	0.3770
***lm(Cancer_Rate ~ Δ9THC)***						
Δ9THC	0.33 (0.15, 0.5)	0.0002	0.0169	13.8	1748	0.0002
lm(Cancer_Rate ~ Exposure * Drug)						
Drug_Rate: Cannabis	4.63 (2.11, 7.15)	0.0003	0.0207	9.82	93,740	5.39E-15
Drug_Rate: Alcohol	−3.22 (−6.21, −0.22)	0.0356				
Drug_Rate: Analgesics	−6.63 (−10.51, −2.75)	0.0008				
Cocaine	−1.06 (− 1.63, −0.49)	0.0003				
Cannabis	−1.32 (− 1.89, −0.74)	0.0000				
Drug_Rate	−3.63 (−4.86, −2.4)	0.0000				
lm(Cancer_Rate ~ Exposure * Cannabinoid)						
Cannabinol	6.54 (5.07, 8.01)	< 2E-16	0.0402	18.9	72,992	< 2E-16
Cannabigerol	7.65 (5.91, 9.38)	< 2E-16				
Drug_Rate	2.14 (1.55, 2.73)	0.0000				
Cannabichromene	3.86 (0.29, 7.42)	0.0340				
Drug_Rate: Cannabichromene	−3.02 (−5.4, −0.63)	0.0130				
lm(Cancer_Rate ~ Ethnic_THC_Exposure * Ethnicity)						
Ethnic_THC_Exposure	0.14 (0.07, 0.21)	0.0001	0.0021	2.57	64,493	0.0174
Asian-Am_THC_Exposure	0.28 (0.02, 0.55)	0.0360				

**Fig. 7:**
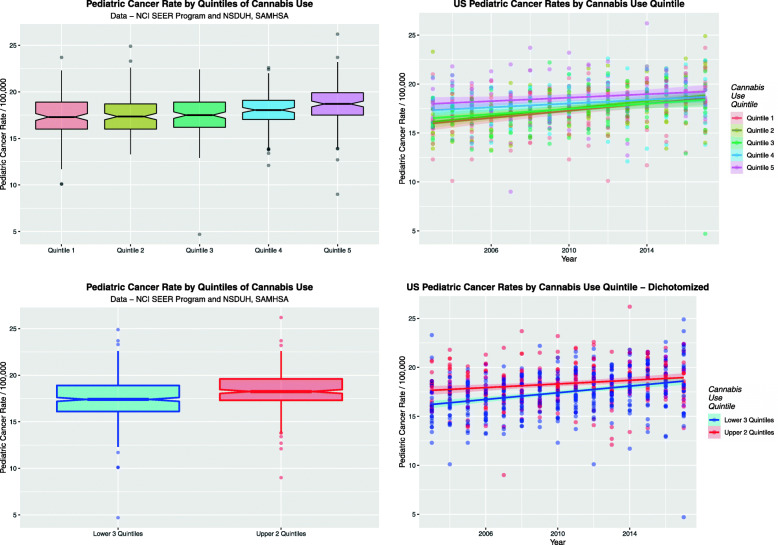
Total pediatric cancer incidence rate by cannabis use quintiles. **a** Boxplot over aggregated time. **b** Scatterplot over time by cannabis use quintiles. **c** Boxplot by dichotomized cannabis use quintiles, highest two quintiles vs. the lowest three. Note non-over-lapping notches indicating significant differences. **d** Scatterplot over time of total pediatric cancer incidence rate by dichotomized cannabis use quintiles

Figure 7 shows the result of assessing the TPCIR as a function of cannabis use quintiles both cross-sectionally (boxplots) and over time (scatterplots). Panel A appears to show a rising trend with cannabis use quintile. One notes in particular that the notches of the fourth and fifth quintiles do not overlap those of Quintiles 1 and 2 which indicates significance. In Panel B the highest two quintiles seem to be above the lower ones over time. Panel C and D look at the data dichotomized into the two highest quintiles compared to the three lower ones. Again in Panel C it is clear that the notches of the upper quintiles do not overlap those of the lower ones. Panel D shows that this holds true over time. Raw mean quintile data with standard errors appears in Supplementary Table 1.

When comparing the highest and lowest quintile of cannabis use the TPCIR in the highest quintiles is significantly greater than that in the lowest quintile (t = 5.038, df = 299.6, *P* = 8.15 × 10^− 7^). Comparing the two dichotomized cannabis quintile groups they are also significantly different (t = 5.641, df = 673.6, *P* = 2.4810^− 8^). The chi squared test for trend across the quintiles does not reach significance (Chi.Squ. = 465.4, df = 420, *P* = 0.0623). When these data are examined by linear regression the significant results shown in Table 3 are found.

**Table 3:** Linear Regressions on Quintiles

Parameter Estimates			Model Parameters			
Parameter	Estimate (C.I.)	***P***-Value	R-Squared	F	dF	P-Value
***Quintiles***						
***lm(Cancer_Rate ~ Quintile)***						
Quintile 2	0.2 (−0.29, 0.69)	0.4242	0.04527	9.34	4745	2.27E-07
Quintile 3	0.14 (−0.35, 0.63)	0.5655				
Quintile 4	0.72 (0.23, 1.2)	0.0042				
Quintile 5	1.31 (0.82, 1.8)	1.8E-07				
***Dichotomized Quintiles***						
***lm(Cancer_Rate ~ Dichotomized_Quintiles)***						
Upper_2_Quintiles	0.9 (0.58, 1.22)	3.9E-08	0.0383	30.9	1748	3.86E-08
***Dichotomized Quintiles Over Time***						
***lm(Cancer_Rate ~ Year + Dichotomized_Quintiles)***						
Upper_2_Quintiles	0.9 (0.59, 1.2)	1.1E-08	0.111	47.8	2747	< 2E-16
***lm(Cancer_Rate ~ Year***: ***Dichotomized_Quintiles)***						
Lower_3_Quintiles	0.139 (0.1, 0.17)	1.2E-14	0.111	47.7	2747	< 2E-16
Upper_2_Quintiles	0.1395 (0.1, 0.17)	1.0E-14				

Table 4 presents results from increasingly complex robust inverse probability weighted marginal structural models. Results for additive, interactive with drugs only, interactive including drugs, race and income and interactive including cannabinoids, drugs, race and income models are shown. It is particularly noteworthy that in a simple additive robust model (listed first in the table) cannabis is independently highly significant (β-estimate = 9.55 95%C.I. (3.95, 15.15), *P* = 0.0016).

**Table 4:** Robust Generalized Linear Regression Models

Parameter	Estimate (C.I.)	P-Value
***Additive Model***		
***svyglm(Cancer_Rate ~ Cigarettes + Cannabis + Analgesics + Alcohol + Cocaine)***		
Cannabis	9.55 (3.95, 15.15)	0.0016
Alcohol	−19.69 (−27.68, − 11.7)	1.5E-05
***Interactive Model***		
svyglm(Cancer_Rate ~ Cigarettes * Cannabis * Analgesics * Alcohol + Cocaine)		
Cigarettes: Cannabis: Analgesics	268.42 (91.87, 444.96)	0.0046
Cigarettes: Analgesics	−59.54 (−92.24, −26.84)	0.0009
***Full Interactive Model***		
svyglm(Cancer_Rate ~ Cigarettes * Cannabis * Analgesics * Alcohol + Cocaine + 6_Races + Income)		
White	8.1 (6.04, 10.17)	4.2E-09
Hispanic	0.74 (0.37, 1.11)	0.0004
Asian	0.77 (0.38, 1.16)	0.0004
Cigarettes: Alcohol: Analgesics	331.59 (121.58, 541.61)	0.0038
Cigarettes: Cannabis: Analgesics	2537.45 (833.94, 4240.95)	0.0060
Cigarettes	52.94 (15.62, 90.27)	0.0086
Alcohol: Analgesics	871 (196.96, 1545.04)	0.0158
Cannabis: Alcohol	543.49 (110.37, 976.6)	0.0189
Cigarettes: Cannabis	−268.79 (− 471.82, −65.76)	0.0136
Alcohol	− 119.12 (−207.37, −30.87)	0.0120
Cannabis: Alcohol: Analgesics	− 4989.69 (− 8616.76, − 1362.61)	0.0106
AIAN	−6.66 (− 11.36, − 1.95)	0.0087
Cigarettes: Analgesics	−500.17 (− 808.72, − 191.63)	0.0030
***Full Interactive Model with Cannabinoids***		
svyglm(Cancer_Rate ~ Cigarettes * Δ9THC * Cannabigerol * Alcohol + Analgesics + Cocaine + 6_Races + Income)		
White	7.87 (5.73, 10.02)	1.9E-08
Cocaine	25.98 (12.75, 39.21)	0.0005
Asian	0.68 (0.31, 1.06)	0.0010
Hispanic	0.59 (0.23, 0.94)	0.0026
Cigarettes: Δ9THC: Analgesics	34.32 (13.53, 55.11)	0.0026
Cigarettes: Cannabigerol	270.35 (104.12, 436.59)	0.0030
Cigarettes: Δ9THC	2.93 (0.58, 5.28)	0.0195
Δ9THC: Cannabigerol	29.24 (4.95, 53.53)	0.0239
AIAN	−5.96 (−11.16, −0.75)	0.0311
Cigarettes: Δ9THC: Alcohol	−13.34 (− 24.92, − 1.77)	0.0300
Cigarettes: Δ9THC: Cannabigerol	− 103.55 (− 181.75, − 25.34)	0.0136
Cannabigerol	− 115.2 (− 189.34, −41.06)	0.0043
Cigarettes: Analgesics	−87.59 (− 127.51, − 47.66)	1.2E-04

Since these robust models are not accompanied by a model variance it is necessary to also use a mixed effects model system in order to be able to calculate e-Values subsequently. Mixed effects modelling was also conducted after inverse probability weighting (Table 5). Again a series of increasingly complex models is shown progressing through additive, drug-interactive, full models including drugs, income and ethnicity, and a full model including the two cannabinoids THC and cannabigerol. Importantly in the first three models cannabis is independently highly statistically significant (from β-estimate = 79.27 (56.77, 101.78), *P* = 1.2 × 10^− 11^).

**Table 5:** Mixed Effects Regression Models

Parameters			Model Parameters			
Parameter	Estimate (C.I.)	***P***-Value	SD	AIC	BIC	logLik
***Additive Model***						
***lme(Cancer_Rate ~ Cigarettes + Cannabis + Analgesics + Alcohol + Cocaine)***						
Cannabis	5.34 (0.07, 10.6)	0.0472	3.43138	3884.77	3912.46	− 1936.39
Analgesics	−11.02 (− 18.65, −3.39)	0.0048				
***Interactive Model***						
lme(Cancer_Rate ~ Cigarettes * Cannabis * Analgesics * Alcohol + Cocaine)						
Cannabis	72.88 (49.6, 96.15)	1.4E-09	3.31033	3781.12	3836.4	− 1878.56
Cigarettes	43.36 (27.68, 59.04)	8.2E-08				
Alcohol: Analgesics	1523.99 (970.61, 2077.38)	9.3E-08				
Cigarettes: Cannabis: Analgesics	2788.19 (1676.17, 3900.2)	1.1E-06				
Cannabis: Alcohol: Analgesics	− 4554.93 (− 6709.17, − 2400.69)	3.8E-05				
Cigarettes: Analgesics	−539.08 (− 790.18, − 287.99)	2.9E-05				
Analgesics	−87.43 (−121.63, −53.23)	6.9E-07				
Alcohol	− 82.06 (− 113.58, −50.54)	4.3E-07				
Cigarettes: Cannabis	−284.5 (−376.55, − 192.45)	2.3E-09				
***Full Interactive Model***						
lme(Cancer_Rate ~ Cigarettes * Cannabis * Analgesics * Alcohol + Cocaine + 6 Races + Income)						
White	11.8 (8.45, 15.14)	1.1E-11	3.18221	3715.57	3784.61	− 1842.79
Cannabis	79.27 (56.77, 101.78)	1.2E-11				
Asian	2.54 (1.8, 3.27)	2.6E-11				
Cigarettes: Alcohol: Analgesics	1636.35 (1108.24, 2164.46)	2.1E-09				
Cigarettes	45.74 (30.44, 61.04)	7.2E-09				
Cigarettes: Cannabis: Analgesics	2525.7 (1488.65, 3562.75)	2.2E-06				
Alcohol: Analgesics	959.4 (425.8, 1493)	4.5E-04				
Cannabis: Alcohol: Analgesics	− 4264.85 (− 6314.08, − 2215.61)	5.1E-05				
Alcohol	−93.44 (− 124.44, −62.43)	5.5E-09				
Cigarettes: Analgesics	−766.56 (− 1011.12, −521.99)	1.4E-09				
Cigarettes: Cannabis	−290.63 (− 373.83, −207.42)	1.7E-11				
Income	−9.44 (−12.02, −6.87)	1.7E-12				
***Full Interactive Model with Cannabinoids***						
lme(Cancer_Rate ~ Cigarettes * Δ9THC * Cannabigerol * Alcohol + Analgesics + Cocaine + 6 Races + Income)						
White	15.39 (11.82, 18.96)	1.8E-16	3.16296	3743.28	3798.56	− 1859.64
Asian	2.46 (1.76, 3.16)	1.2E-11				
Cigarettes: Cannabigerol: Alcohol	4741.19 (3077.86, 6404.51)	3.3E-08				
Cigarettes: Δ9THC	26.57 (15.54, 37.6)	2.8E-06				
Δ9THC: Alcohol	14.95 (7.74, 22.16)	5.4E-05				
Hispanic	0.7 (0.14, 1.26)	1.4E-02				
Cigarettes: Cannabigerol	− 663.69 (− 971.24, −356.13)	2.7E-05				
Income	−7.76 (−10.11, −5.41)	1.9E-10				
Cigarettes: Δ9THC: Alcohol	−240.65 (− 304.57, − 176.72)	4.6E-13				

Since the data are gridded in space and time they are well suited for panel linear modelling, a technique which, in addition to inverse probability weighting, allows the added refinements of instrumental variables and temporal lagging. Temporal lagging is pathophysiologically important in such studies as it is likely that any procarcinogenic or environmental exposure takes some time to work before the clinical and epidemiological impact of genotoxicity becomes evident. Again a series of increasingly complex models is presented at increasing lags (Table 6). Cannabis is again highly significant in many terms, including being independently significant in additive models (from β-estimate = 5.31 (1.68, 8.95), *P* = 0.0042).

**Table 6:** Panel Regression Models

Model Specification		Parameters			Model Parameters				
Instrumental Variables	Lagged Parameter	Parameter	Estimate (C.I.)	***P***-Value	Adj. R-Squared	Chi.Squ.	F	dF	P
		***Additive model***							
		***plm(Cancer_Rate ~ Cigarettes + Cannabis + Analgesics + Alcohol + Cocaine)***							
		Cannabis	5.31 (1.68, 8.95)	0.0042	0.0790	80.6858		3	< 2.2E-16
		Analgesics	−9.3 (−14.93, −3.67)	0.0012					
		Cigarettes	−4.53 (−7.15, −1.92)	0.0007					
		***Interactive model***							
		plm(Cancer_Rate ~ Cigarettes * Cannabis * Analgesics * Alcohol + Cocaine)							
		Cigarettes: Cannabis	24.47 (11.37, 37.57)	0.0003	0.0663	83.1987		4	< 2.2E-16
		Cocaine	−11.93 (−22.84, −1.02)	0.0321					
		Analgesics	−8.25 (−14.2, − 2.3)	0.0066					
		Cigarettes	−5.83 (−8.92, −2.73)	0.0002					
		***Interactive Full model***							
		plm(Cancer_Rate ~ Cigarettes * Cannabis * Analgesics * Alcohol + Cocaine + 6_Races + Income)							
		White	8.57 (7.18, 9.96)	< 2.2E-16	0.1927	23.5694		13,736	< 2.2E-16
		Asian	0.92 (0.67, 1.17)	2.0E-12					
		Hispanic	0.71 (0.47, 0.94)	7.1E-09					
		Cigarettes	69.38 (39.16, 99.59)	7.9E-06					
		Cigarettes: Alcohol: Analgesics	1169.92 (598.17, 1741.68)	6.7E-05					
		Cannabis: Alcohol	719.64 (352.45, 1086.83)	1.3E-04					
		Cigarettes: Cannabis: Analgesics	2926.99 (1407.72, 4446.25)	0.0002					
		Analgesics	58.43 (22.7, 94.17)	0.0014					
		Alcohol: Analgesics	709.96 (139.61, 1280.31)	0.0149					
		Cannabis: Alcohol: Analgesics	− 5916.9 (− 9125.97, − 2707.83)	0.0003					
		Cigarettes: Cannabis	− 345.82 (−521.51, − 170.13)	1.2E-04					
		Alcohol	− 153.51 (−219.89, −87.13)	6.8E-06					
		Cigarettes: Analgesics	− 716.53 (− 1007.91, −425.15)	1.8E-06					
		***Interactive Full model - 2 Lags***							
	Cigarettes, 2	plm(Cancer_Rate ~ Cigarettes * Cannabis * Analgesics * Alcohol + Cocaine + 6_Races + Income)							
	Cannabis, 2	White	9.28 (7.88, 10.67)	< 2.2E-16	0.2014		33.9397	7642	2.0E-01
	Analgesics, 2	Asian	0.96 (0.71, 1.22)	2.1E-13					
	Alcohol, 2	Hispanic	0.67 (0.41, 0.94)	9.4E-07					
	Cocaine, 2	Cigarettes	18.29 (9.29, 27.29)	7.6E-05					
		Cigarettes: Alcohol: Analgesics	513.89 (96.6, 931.17)	0.0161					
		Cigarettes: Analgesics	−92.64 (−154.24, −31.03)	0.0033					
		Cigarettes: Alcohol	− 95.72 (− 146.79, −44.65)	0.0003					
		***Interactive Full model - 4 Lags***							
		plm(Cancer_Rate ~ Cigarettes * Cannabis * Analgesics * Alcohol + Cocaine + 6_Races + Income)							
	Cigarettes, 4	White	8.71 (6.97, 10.46)	< 2.2E-16	0.1990		17.6055	12,537	< 2.2E-16
	Cannabis, 4	Hispanic	0.85 (0.54, 1.16)	7.3E-08					
	Analgesics, 4	Asian	0.72 (0.4, 1.05)	1.7E-05					
	Alcohol, 4	Cigarettes: Cannabis	233.66 (115.14, 352.18)	1.3E-04					
	Cocaine, 4	Cigarettes: Alcohol: Analgesics	1975.86 (855.19, 3096.52)	0.0006					
		Cannabis: Alcohol: Analgesics	1972.91 (573.99, 3371.83)	0.0059					
		Alcohol	105.37 (17.01, 193.73)	0.0198					
		AIAN	−8.33 (−14.85, −1.8)	0.0127					
		Alcohol: Analgesics	− 647.54 (− 1104.4, − 190.68)	0.0057					
		Cannabis: Alcohol	−376.97 (−619.06, − 134.87)	0.0024					
		Cigarettes: Alcohol	−286.48 (− 462.48, −110.48)	0.0015					
		Cigarettes: Cannabis: Analgesics	− 1300.25 (−2003.68, − 596.83)	0.0003					
		***Interactive Full model - 5 Lags***							
		plm(Cancer_Rate ~ Cigarettes * Cannabis * Analgesics * Alcohol + Cocaine + 6_Races + Income)							
THC	Cigarettes, 5	White	6.7228 (5.29, 8.15)	< 2.2E-16	0.2302	233.988		10	< 2.2E-16
Cannabigerol	Cannabis, 5	Hispanic	0.652 (0.41, 0.89)	7.2E-08					
Cannabinol	Analgesics, 5	Asian	0.6755 (0.42, 0.93)	2.6E-07					
Cannabichromene	Alcohol, 5	Cigarettes: Cannabis	28.717 (14.28, 43.16)	0.0001					
	Cocaine, 5	Cigarettes: Cannabis: Alcohol: Analgesics	8122.4789 (3192.57, 13,052.39)	0.0012					
		Cannabis: Analgesics	463.7354 (130.9, 796.57)	0.0063					
		Cigarettes: Cannabis: Analgesics	− 1099.2482 (−1927.82, −270.68)	0.0093					
		Cannabis: Alcohol: Analgesics	− 3523.9404 (− 5495.28, − 1552.6)	0.0005					
		AIAN	−9.4157 (−14.03, −4.81)	0.0001					
		Cigarettes	−5.1117 (−7.44, −2.78)	1.7E-05					
		***Interactive Full Model with Racial Cannabis Exposure as Instrumental Variables***							
THC Exposure		plm(Cancer_Rate ~ Cigarettes * Cannabis * Analgesics * Alcohol + Cocaine + 6_Races + Income)							
In:		White	0.7294 (−11.72, 25.62)	< 2.2E-16	0.2300	232.721		9	< 2.2E-16
Caucas-Am.		Asian	0.1306 (−9.77, 11.17)	9.2E-08					
African-Am.		Hispanic	0.1202 (−9.65, 10.91)	1.6E-07					
Hispan-Am.		Cigarettes: Cannabis: Alcohol: Analgesics	731.4135 (3112.74, 3129.47)	2.0E-05					
Asian-Am.		Cannabis: Analgesics	35.5327 (136.48, 152.42)	4.8E-05					
AIAN-Am.		Cocaine	4.9629 (7.98, 18.41)	0.0078					
NHPI-Am.		AIAN	2.3266 (−1.45, −16.93)	7.9E-05					
		Cannabis: Alcohol: Analgesics	378.1363 (− 1752.55, − 1770.82)	3.2E-06					
		Cigarettes: Analgesics	9.4453 (−37.01, −56.39)	7.7E-07					

**Fig. 8:**
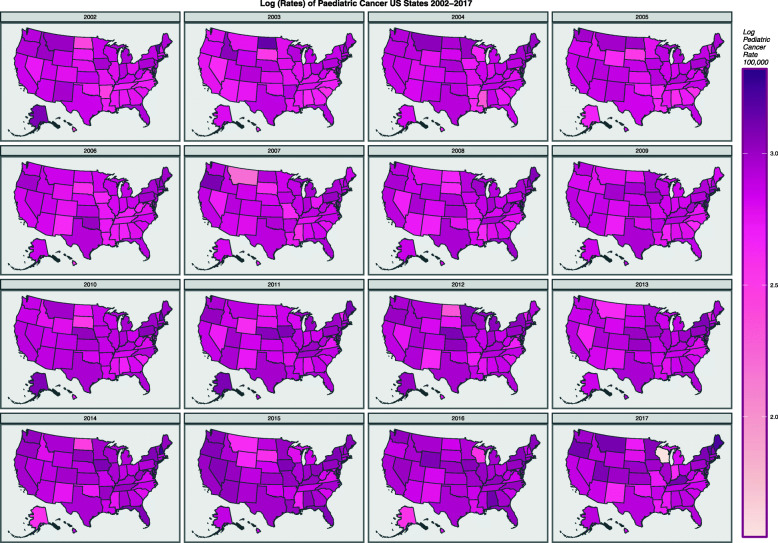
Map graph of total pediatric cancer incidence rate by state over time sequence, by year

**Fig. 9:**
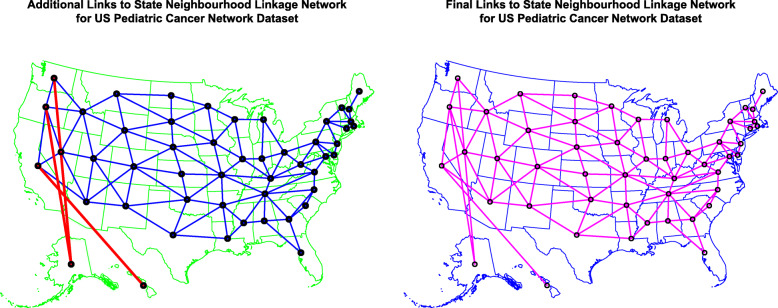
Geospatial linkages used for geospatiotemporal regression analyses. Note Alaska and Hawaii elided arithmetically onto continental USA. **a** Edited spatial links. **b** Final links

Data is also evidently oriented in space and time and is thus eminently suited for formal spatiotemporal analysis. Map-graphs of the data over the 16 years 2002–2017 are shown in Fig. 8. Fig. 9 shows the geospatial relationships between the contiguous American states and the manner in which links to Hawaii and Alaska have been edited in to define the final spatial neighbourhood network based on “queen” (edge and corner) contiguity. This neighbourhood sparse weights matrix is utilized in all the spatial regressions which follow.

Table 7 shows the initial results from a series of additive and increasingly complex unlagged interactive spatiotemporal models. The table includes the log of the maximum likelihood ratio (Log.Lik.) at model optimization, and the specifically geospatial model coefficients phi, psi, rho and lambda (see Methods). Since all four of these parameters are generally highly significant this confirms that the full model specification (denoted ‘sem2srre’ in splm::spreml) is appropriate. The Table also lists the standard deviation of each model which is a required input for E-Value calculation. Again cannabis is noted to be independently highly significant in each model.

**Table 7:** Introductory Spatiotemporal Models

Parameter			Model				
Parameter	Estimate (C.I.)	P-Value	LogLik	S.D.	Model Parameter	Estimate	P-Value
***Additive Model***							
***spreml(Cancer_Rate ~ Cigarettes + Cannabis + Alcohol + Analgesics + Cocaine)***							
Cannabis	5.16 (2.26, 8.06)	0.0005	− 1541.00	1.9451	phi	0.3170	0.0002
Analgesics	− 4.6 (−9.18, − 0.02)	0.0490			psi	0.1480	0.0007
Cigarettes	−2.72 (−4.85, −0.59)	0.0124			rho	−0.4959	2.2E-05
					lambda	0.4598	8.2E-08
***3-Way Interactive model***							
spreml(Cancer_Rate ~ Cigarettes * Cannabis * Alcohol + Analgesics + Cocaine)							
Cannabis	20.68 (7.02, 34.33)	0.0030	−1541.24	1.9495	phi	0.3466	0.0002
Cigarettes: Alcohol	48.6 (2.75, 94.46)	0.0378			psi	0.1488	0.0006
Cigarettes: Cannabis	−46.18 (−84.76, −7.6)	0.0190			rho	−0.5248	2.4E-06
Alcohol	−25.69 (−44.01, −7.37)	0.0060			lambda	0.4837	1.3E-09
***4-Way Interactive model***							
spreml(Cancer_Rate ~ Cigarettes * Cannabis * Alcohol * Analgesics + Cocaine)	phi	0.3169			0.0002		
Cannabis	5.42 (2.34, 8.5)	0.0006	− 1540.34	1.9470	psi	0.1479	0.0007
Alcohol	−8.18 (−14.61, −1.74)	0.0128			rho	−0.4896	3.1E-05
Cigarettes: Analgesics	−31.81 (−56.07, −7.54)	0.0102			lambda	0.4514	2.1E-07
***Interactive Full Model - 0 Lags***							
spreml(Cancer_Rate ~ Cigarettes * Cannabis * Alcohol * Analgesics + Cocaine + 6_Races + Income)							
Cigarettes	28.41 (12.48, 44.34)	0.0005	− 1520.55	1.8458	phi	0.1709	0.0017
Cannabis	45.67 (18.77, 72.56)	0.0009			psi	0.1079	0.0138
White	5.24 (3.38, 7.1)	0.0000			rho	−0.4106	0.0029
Cigarettes: Cannabis: Alcohol	840.86 (416.29, 1265.44)	0.0001			lambda	0.3643	0.0006
Alcohol: Analgesics	638.1 (283.09, 993.12)	0.0004					
Asian-American	0.6 (0.23, 0.97)	0.0015					
Hispanic-American	0.45 (0.11, 0.79)	0.0089					
Cigarettes: Cannabis: Analgesics	966.38 (184.69, 1748.06)	0.0154					
AIAN-American	−8.3 (−15.42, −1.18)	0.0224					
Cigarettes: Analgesics	−240.1 (−391.42, − 88.77)	0.0019					
Cannabis: Alcohol: Analgesics	− 2613.19 (− 4248.03, − 978.35)	0.0017					
Cigarettes: Cannabis	− 235.06 (− 381.19, −88.92)	0.0016					
Alcohol	−79.04 (− 114.93, − 43.14)	1.6E-05					

Table 8 shows the results of models lagged first just with cannabis and then for all drugs. Interactive terms including cannabis continue to be highly significant. Interactive terms including cannabis are significant from β-estimate = 658.72 (396.60, 920.84), *P* = 8.40 × 10^− 7^ for cigarettes: cannabis: alcohol interaction at 2 years of lag.

**Table 8:** Time-Lagged Spatiotemporal Models

Lagged Variables	Parameter			Model				
	Parameter	Estimate (C.I.)	***P***-Value	LogLik	S.D.	Model Parameter	Estimate	***P***-Value
Cannabis, 2	***Full model - 2 Lags - Just Lagging Cannabis***							
	spreml(Cancer_Rate ~ Cigarettes * Cannabis * Alcohol * Analgesics + Cocaine + 6_Races + Income)							
	Caucasian-American	5.3 (3.63, 6.97)	5.3E-10	− 1329.42	1.8583	phi	0.1690	0.0037
	Asian-American	0.63 (0.31, 0.95)	1.3E-04			psi	0.1476	0.0018
	Hispanic-American	0.54 (0.21, 0.86)	0.0013			rho	−0.4435	8.3E-04
	AIAN-American	−11.33 (−18.34, −4.32)	0.0015			lambda	0.4234	9.1E-06
Cannabis, 4	***Full model - 4 Lags - Just Lagging Cannabis***							
	spreml(Cancer_Rate ~ Cigarettes * Cannabis * Alcohol * Analgesics + Cocaine + 6_Races + Income)							
	Caucasian-American	4.81 (2.92, 6.7)	6.1E-07	− 1130.71	1.8616	phi	0.2095	0.0031
	Asian-American	0.67 (0.31, 1.03)	0.0003			psi	0.1134	0.0356
	Hispanic-American	0.55 (0.18, 0.92)	0.0037			rho	−0.5410	3.0E-05
	Cigarettes: Cannabis: Analgesics	261.1 (19.06, 503.15)	0.0345			lambda	0.4597	9.1E-07
	Cannabis: Analgesics	−107.34 (−193.09, −21.6)	0.0141					
	AIAN-American	−12.1 (− 19.56, −4.64)	0.0015					
Cannabis, 6	***Full model - 6 Lags - Just Lagging Cannabis***							
	spreml(Cancer_Rate ~ Cigarettes * Cannabis * Alcohol * Analgesics + Cocaine + 6_Races + Income)							
	Caucasian-American	7.54 (3.96, 11.12)	3.6E-05	−936.96	1.9697	phi	0.2705	0.0022
	Asian-American	0.95 (0.34, 1.54)	0.0020			psi	0.0992	0.1012
	Cannabis	8.49 (1.47, 15.5)	0.0177			rho	0.4222	0.0006
	Hispanic-American	0.7 (0.11, 1.29)	0.0202			lambda	−0.4083	0.0059
	Cannabis: Analgesics	−47.05 (−79.05, −15.03)	0.0040					
	***Full Model - 1 Temporal Lag***							
	spreml(Cancer_Rate ~ Cigarettes * Cannabis * Alcohol * Analgesics + Cocaine + 6_Races + Income)							
Cigarettes, 1	Caucasian-American	5.42 (3.72, 7.12)	4.6E-10	− 1426.33	1.8466	phi	0.1684	0.0027
Alcohol, 1	Asian-American	0.67 (0.33, 1)	0.0001			psi	0.1408	0.0016
Cannabis, 1	Hispanic-American	0.56 (0.22, 0.9)	0.0014			rho	−0.4380	0.0009
Analgesics, 1	Cannabis	7.88 (1.7, 14.06)	0.0125			lambda	0.4226	1.2E-05
Cocaine, 1	Cigarettes: Cannabis: Alcohol	182.23 (29.55, 334.9)	0.0193					
	AIAN-American	−9.03 (−16.1, −1.96)	0.0123					
	Cannabis: Alcohol	− 114.28 (− 198.3, −30.27)	0.0077					
	***Full Model - 2 Temporal Lags***							
	spreml(Cancer_Rate ~ Cigarettes * Cannabis * Alcohol * Analgesics + Cocaine + 6_Races + Income)							
Cigarettes, 2	Caucasian-American	9.62 (6.82, 12.43)	1.7E-11	− 1317.36	1.8519	phi	0.1408	0.0083
Alcohol, 2	Cigarettes: Cannabis: Alcohol	658.72 (396.6, 920.84)	8.4E-07			psi	0.1469	0.0018
Cannabis, 2	Asian-American	1.32 (0.75, 1.89)	6.4E-06			rho	0.3276	0.0126
Analgesics, 2	Alcohol: Analgesics	306.67 (143.27, 470.07)	0.0002			lambda	−0.2888	0.0462
Cocaine, 2	Hispanic-American	0.69 (0.26, 1.12)	0.0016					
	Income	−2.15 (−4.22, −0.08)	0.0415					
	Cannabis: Alcohol: Analgesics	− 1810.02 (− 2618.86, − 1001.18)	1.2E-05					
	Cigarettes: Alcohol	− 133.02 (−184.5, −81.54)	4.1E-07					
	***Full Model - 4 Temporal Lags***							
	spreml(Cancer_Rate ~ Cigarettes * Cannabis * Alcohol * Analgesics + Cocaine + 6_Races + Income)							
Cigarettes, 4	Caucasian-American	5.25 (3.17, 7.33)	7.6E-07	− 1129.73	1.8795	phi	0.1863	0.0058
Alcohol, 4	Cigarettes: Cannabis: Alcohol	472.69 (145.49, 799.88)	0.0046			psi	0.1341	0.0127
Cannabis, 4	Asian-American	0.56 (0.16, 0.95)	0.0055			rho	−0.4598	0.0040
Analgesics, 4	Hispanic-American	0.5 (0.13, 0.87)	0.0085			lambda	0.4021	8.6E-04
Cocaine, 4	Cigarettes: Alcohol: Analgesics	603.85 (143.88, 1063.82)	0.0101					
	Cigarettes: Alcohol	−80.89 (−138.89, −22.89)	0.0063					
	AIAN-American	− 10.99 (− 18.8, −3.18)	0.0058					
	Cigarettes: Cannabis: Alcohol: Analgesics	− 3668.28 (− 6170.15, − 1166.42)	0.0041					
Cigarettes, 6	***Full Model - 6 Temporal Lags***							
	spreml(Cancer_Rate ~ Cigarettes * Cannabis * Alcohol * Analgesics + Cocaine + 6_Races + Income)							
Alcohol, 6	Caucasian-American	4.28 (2.17, 6.4)	7.4E-05	− 938.093	1.9015	phi	0.2238	0.0053
Cannabis, 6	Asian-American	0.5 (0.13, 0.87)	0.0089			psi	0.1218	0.0448
Analgesics, 6	Hispanic-American	0.51 (0.12, 0.91)	0.0115			rho	− 0.5495	7.7E-05
Cocaine, 6	AIAN-American	−11.64 (−19.61, −3.66)	0.0042			lambda	0.5042	1.8E-07

Table 9 presents results of models lagged in space for cannabis and in time for the other drugs.

**Table 9:** Spatially- and Temporally- Lagged Spatiotemporal Models

Lagged Variables	Parameter			Model				
	Parameter	Estimate (C.I.)	***P***-Value	LogLik	S.D.	Model Parameter	Estimate	***P***-Value
	***Full Model - 1 Spatial & 1 Temporal Lag***							
	spreml(Cancer_Rate ~ Cigarettes * Cannabis * Alcohol * Analgesics + Cocaine + 6_Races + Income)							
Cigarettes, 1	Caucasian-American	4.49 (2.56, 6.41)	4.9E-06	− 1422.64	1.8639	phi	0.1534	0.0041
Alcohol, 1	Hispanic-American	0.61 (0.26, 0.96)	0.0006			psi	0.1284	0.0043
Cannabis, Sp1	Cannabis: Analgesics	110.36 (37.53, 183.19)	0.0030			rho	−0.3379	0.0408
Analgesics, 1	Cigarettes: Cannabis: Alcohol	1688.83 (336.9, 3040.77)	0.0143			lambda	0.3229	0.0134
Cocaine, 1	Asian-American	0.46 (0.09, 0.83)	0.0146					
	Cannabis: Alcohol: Analgesics	− 885.51 (− 1625.8, − 145.21)	0.0191					
	AIAN-American	−10.01 (−17.08, −2.94)	0.0055					
	Analgesics	−18.96 (−29.31, − 8.61)	0.0003					
	***Full Model - 2 Spatial & 2 Temporal Lags***							
	spreml(Cancer_Rate ~ Cigarettes * Cannabis * Alcohol * Analgesics + Cocaine + 6_Races + Income)							
Cigarettes, 2	Caucasian-American	8.03 (5.67, 10.39)	2.6E-11	− 1319.97	1.8579	phi	0.0990	0.0324
Alcohol, 2	Asian-American	1.02 (0.53, 1.51)	5.2E-05			psi	0.1426	0.0032
Cannabis, Sp2	Hispanic-American	0.66 (0.33, 0.99)	9.0E-05			rho	−0.2287	0.3086
Analgesics, 2	Analgesics	55.5 (26.18, 84.81)	0.0002			lambda	0.2307	0.2080
Cocaine, 2	Cigarettes: Cannabis: Alcohol: Analgesics	3954.04 (1565.01, 6343.08)	0.0012					
	Cocaine	15.51 (1.58, 29.44)	0.0291					
	Cigarettes: Cannabis: Analgesics	− 749.24 (− 1219.42, − 279.07)	0.0018					
	Alcohol: Analgesics	−377.69 (−553.03, − 202.35)	2.4E-05					
	***Full Model - 4 Spatial & Temporal Lags***							
	spreml(Cancer_Rate ~ Cigarettes * Cannabis * Alcohol * Analgesics + Cocaine + 6_Races + Income)							
Cigarettes, 4	Caucasian-American	5.18 (3.29, 7.07)	7.7E-08	− 1133.35	1.8790	phi	0.1850	0.0049
Alcohol, 4	Asian-American	0.59 (0.25, 0.93)	0.0008			psi	0.1286	0.0176
Cannabis, Sp4	Hispanic-American	0.52 (0.16, 0.87)	0.0045			rho	−0.4868	0.0004
Analgesics, 4	Alcohol: Analgesics	−27.25 (−54.07, − 0.43)	0.0464			lambda	0.4290	2.5E-05
Cocaine, 4	AIAN-American	−10.96 (−18.4, −3.51)	0.0039					

Table 10 presents the results of temporally lagged interactive space-time models including the two cannabinoids THC and cannabigerol. Cannabigerol is independently significant at 2 lags, and the THC:cannabigerol interaction is significant at zero, two and six lags.

**Table 10:** Spatially- and Temporally- Lagged Spatiotemporal Models

Lagged Variables	Parameter			Model				
	Parameter	Estimate (C.I.)	***P***-Value	LogLik	S.D.	Model Parameter	Estimate	***P***-Value
	***Cannabinoids***							
	***Cannabinoids as Main Effects***							
	spreml(Cancer_Rate ~ Cigarettes * THC * Cannabigerol * Alcohol + Analgesics + Cocaine)							
	Caucasian-American	4.83 (2.77, 6.89)	4.5E-06	− 1511.96	1.8350	phi	0.2050	0.0009
	Cigarettes: Alcohol	334 (171.12, 496.88)	0.0001			psi	0.0889	0.0450
	Alcohol: Analgesics	312 (149.91, 474.09)	0.0002			rho	−0.4495	0.0003
	Cigarettes: Δ9THC: Analgesics	391 (181.28, 600.72)	0.0003			lambda	0.3639	0.0001
	Δ9THC: Alcohol	116 (51.71, 180.29)	0.0004					
	Cigarettes: Δ9THC: Cannabigerol: Alcohol	4810 (2124.8, 7495.2)	0.0004					
	Δ9THC: Cannabigerol	109 (41.58, 176.42)	0.0016					
	Analgesics	96.5 (35.74, 157.26)	0.0018					
	Asian-American	0.57 (0.19, 0.94)	0.0029					
	Δ9THC: Cannabigerol: Alcohol: Analgesics	5640 (1680.8, 9599.2)	0.0052					
	Hispanic-American	0.41 (0.07, 0.76)	0.0193					
	Cigarettes: Δ9THC	9.01 (0.6, 17.42)	0.0359					
	AIAN-American	−8.84 (−16.13, −1.55)	0.0175					
	Cigarettes: Δ9THC: Cannabigerol: Alcohol: Analgesics	−18,100 (−29,977.6, − 6222.4)	0.0028					
	Cigarettes: Δ9THC: Cannabigerol	− 385 (−612.36, − 157.64)	0.0009					
	Δ9THC: Cannabigerol: Alcohol	−1480 (− 2346.32, −613.68)	0.0008					
	Δ9THC: Analgesics	−130 (− 199.78, −60.22)	0.0003					
	Cigarettes: Δ9THC: Alcohol	− 384 (−583.92, −184.08)	0.0002					
	Cigarettes: Analgesics	−383 (− 563.32, −202.68)	3.2E-05					
	Alcohol	− 137 (−197.96, −76.04)	1.1E-05					
	***Cannabinoids as Main Effects - 2 Lags***							
	spreml(Cancer_Rate ~ Cigarettes * THC * Cannabigerol * Alcohol + Analgesics + Cocaine)							
THC, 2	Caucasian-American	4.63 (2.53, 6.72)	1.5E-05	− 1320.47	1.8880	phi	0.1976	0.0021
Cannabigerol, 2	Cannabigerol	21.6 (9.29, 33.9)	0.0006			psi	0.1322	0.0052
	THC: Alcohol	10.25 (4.12, 16.37)	0.0010			rho	−0.3332	0.0881
	Asian-American	0.56 (0.17, 0.95)	0.0053			lambda	0.3037	0.0500
	Cannabigerol: Alcohol: Analgesics	1176.24 (308.66, 2043.82)	0.0079					
	Hispanic-American	0.47 (0.1, 0.84)	0.0117					
	AIAN-American	−10.84 (−18.51, −3.17)	0.0056					
	Cannabigerol: Alcohol	−288.07 (−474.04, − 102.11)	0.0024					
	THC: Cannabigerol: Analgesics	92.38 (39.25, 145.5)	0.0007					
	***Cannabinoids as Main Effects - 4 Lags***							
	spreml(Cancer_Rate ~ Cigarettes * THC * Cannabigerol * Alcohol + Analgesics + Cocaine)							
THC, 4	Caucasian-American	4.31 (2.26, 6.36)	3.9E-05	− 1126.72	1.8642	phi	0.1876	0.0047
Cannabigerol, 4	Cigarettes: THC	2.87 (1.47, 4.27)	5.9E-05			psi	0.1223	0.0246
	Asian-American	0.64 (0.26, 1.03)	0.0010			rho	−0.4917	0.0007
	Hispanic-American	0.58 (0.21, 0.95)	0.0021			lambda	0.3940	0.0004
	Cigarettes: Cannabigerol: Alcohol	668.38 (191.09, 1145.67)	0.0061					
	Cigarettes	−3.45 (−5.85, −1.04)	0.0050					
	AIAN-American	−11.66 (−19.15, −4.17)	0.0023					
	Cannabigerol: Alcohol	−329.3 (−523.66, −134.95)	0.0009					
	***Cannabinoids as Main Effects - 6 Lags***							
	spreml(Cancer_Rate ~ Cigarettes * THC * Cannabigerol * Alcohol + Analgesics + Cocaine)							
THC, 6	Cigarettes: THC	28.16 (18.61, 37.71)	7.6E-09	− 918.382	1.8922	phi	0.2868	0.0023
Cannabigerol, 6	THC: Cannabigerol	46.22 (30.06, 62.38)	2.1E-08			psi	0.1197	0.0495
	Asian-American	0.67 (0.21, 1.12)	0.0039			rho	−0.5066	0.0004
	Caucasian-American	3.19 (1.01, 5.38)	0.0042			lambda	0.3707	0.0007
	Cocaine	18.22 (3.99, 32.46)	0.0121					
	Cigarettes: Cannabigerol: Alcohol	724.22 (143.74, 1304.71)	0.0145					
	AIAN-American	−10.25 (−18.78, −1.73)	0.0184					
	Cannabigerol: Alcohol	−329.39 (− 580.71, −78.07)	0.0102					
	Cigarettes: THC: Cannabigerol	−177.1897 (−248.1, − 106.28)	9.7E-07					
	THC	−7.21 (−9.86, −4.55)	1.1E-07					
	Cigarettes	−29.01 (−39.21, −18.82)	2.4E-08					

As mentioned in Methods, well described ethnic disparities exist for many tumours including total cancer. However it is important to consider to what extent such drug use disparities might account for the known epidemiology of TPCIR . Table 11 presents an interactive geospatial regression of the TPCIR against THC exposure of five races as indicated with highly significant results.

**Table 11:** Spatially- and Temporally- Lagged Spatiotemporal Models

Parameter			Model				
Parameter	Estimate (C.I.)	***P***-Value	LogLik	S.D.	Model Parameter	Estimate	***P***-Value
***Cancer Incidence as a Function of Racial Cannabis Exposure***							
spreml(Cancer_Rate ~ NHWhite_THC_Exp + NHBlack_THC_Exp * Hispanic_THC_Exp *Asian_THC_Exp * AIAN_THC_Exp)							
Afric-Am._THC_Exp: Hispan.Am_THC_Exp	1.74 (1.18, 2.29)	1.1E-09	− 1532.27	1.9803	phi	0.3887	0.0001
Afric-Am._THC_Exp: Hispan.Am_THC_Exp: Asian-Am._THC_Exp: AIAN-Am._THC_Exp	0.15 (0.09, 0.21)	1.9E-06			psi	0.1542	0.0005
Asian-Am._THC_Exp: AIAN-Am._THC_Exp	0.89 (0.37, 1.41)	0.0008			rho	−0.4676	0.0002
Afric-Am._THC_Exp: Hispan.Am_THC_Exp: Asian-Am._THC_Exp	−1.11 (−1.55, − 0.67)	8.8E-07			lambda	0.4215	8.1E-06
Afric-Am._THC_Exp: Hispan.Am_THC_Exp: AIAN-Am._THC_Exp	−0.2 (− 0.28, − 0.13)	4.8E-08					
Caucasian-American_THC_Exposure	−1.27 (−1.65, −0.89)	5.0E-11					

E-Values are an important way of quantitating the magnitude of co-association required of any unmeasured confounder with both the exposure and outcome variables to explain away the observed effects. Table 12 presents selected E-Value calculations from linear, mixed effects and geospatial models presented in preceding Tables. The key variable to observe is the final number at the right hand side representing the minimum E-Value, and should be read in the light of the observation by one of its originators that E-Values in the literature over approximately 1.25 are considered noteworthy [56]. In general terms the E-Values fall in the sequence geospatial models > mixed effects models > linear models, related partly to the much smaller model variance of more complex models.

**Table 12:** Spatially- and Temporally- Lagged Spatiotemporal Models

Parameter	Estimate (C.I.)	R.R. (C.I.)	E-Values
***LINEAR REGRESSION***			
***Cancer Rate Over Time***			
Year	0.14 (0.1, 0.17)	1.06 (1.04, 1.08)	1.31, 1.27
***Cancer Rate by*** **Δ*9THC***			
Δ9THC	0.33 (0.15, 0.5)	1.15 (1.07, 1.23)	1.55, 1.33
***Cancer Rate by Drug Rate***			
Drug_Rate: Cannabis	4.63 (2.11, 7.15)	6.83 (2.41, 19.41)	13.15, 4.25
***Cancer Rate by Cannabinoid Over Time***			
Cannabinol	6.54 (5.07, 8.01)	15.54 (8.39, 28.78)	30.58, 16.27
Cannabigerol	7.65 (5.91, 9.38)	24.71 (11.96, 51.02)	48.91, 23.41
Drug_Rate	2.14 (1.55, 2.73)	2.45 (1.91, 3.14)	4.34, 3.24
Cannabichromene	3.86 (0.29, 7.42)	5.04 (1.14, 22.44)	9.54, 1.51
***Cancer Rate by Ethnic Cannabis Exposure***			
Ethnic_THC_Exposure	0.14 (0.07, 0.21)	1.06 (1.03, 1.09)	1.31, 1.20
Asian-Am_THC_Exposure	0.28 (0.02, 0.55)	1.12 (1.01, 1.26)	1.50, 1.10
***Legal Status***			
Decriminalized	0.85 (0.44, 1.26)	1.42 (1.20, 1.69)	2..20, 1.69
Liberal	0.663 (0.35, 0.98)	1.32 (1.15, 1.50)	1.96, 1.58
Legal	1.3286 (0.47, 2.19)	1.73 (1.21, 2.45)	2.86, 1.72
***Cancer by Legal Status***			
Decriminalized	0.78 (0.37, 1.19)	1.38 (1.16, 1.64)	2.11, 1.60
Legal	1.51 (0.68, 2.35)	1.87 (1.33, 2.66)	3.16, 1.98
Cancer by Year * Status			
Year: Decriminalized	0.0003 (0.0001, 0.0005)	1.00013 (1.00004, 1.00021)	1.011, 1.006
Cancer by Year * Dichotomized_Status			
Year: Liberal	0.0002 (0, 0.0004)	1.00008 (1.00001, 1.00015)	1.0090, 1.0035
***MIXED EFFECTS REGRESSION***			
***Additive Model***			
Cannabis	5.34 (0.07, 10.6)	4.11 (1.02, 16.59)	7.70, 1.18
***Interactive Drugs Model***			
Cannabis	72.88 (49.6, 96.15)	5.02E+ 08 (8.45E+ 05, 2.97E+ 11)	1.01E+ 09, 1.69E+ 06
Cigarettes: Cannabis: Analgesics	2788.19 (1676.17, 3900.2)	Infinity (2.40E+ 200, Infinity)	Infinity, Infinity
***Full Interactive Model***			
Cannabis	79.27 (56.77, 101.78)	7.00E+ 09 (1.14E+ 07, 4.31E+ 12)	1.40E+ 09, 2.27E+ 07
Cigarettes: Cannabis: Analgesics	2525.7 (1488.65, 3562.75)	Infinity (1.38E+ 185, Infinity)	Infinity, Infinity
***Full Interactive Model with Cannabinoids***			
Cigarettes: Cannabigerol: Alcohol	4741.19 (3077.86, 6404.51)	Infinity (Infinity, Infinity)	Infinity, Infinity
Δ9THC: Alcohol	14.95 (7.74, 22.16)	73.78 (9.31, 584.34)	147.07, 18.12
Cigarettes: Δ9THC	26.57 (15.54, 37.6)	2.09E+ 03 (87.97, 4.95E+ 04)	4.18E+ 03, 175.45
***GEOSPATIAL REGRESSION***			
***Additive Model***			
Cannabis	5.16 (2.26, 8.06)	11.18 (2.89, 43.30)	21.84,. 5.22
***3-Way Interactive model***			
Cannabis	20.68 (7.02, 34.33)	1.55E+ 04 (26.85, 9.01E+ 06)	3.11E+ 04, 53.19
***4-Way Interactive model***			
Cannabis	5.42 (2.34, 8.5)	12.61 (2.99, 53.07)	24.71, 5.45
***Interactive Full Model - 0 Lags***			
Cannabis	45.67 (18.77, 72.56)	6.00E+ 10 (1.07E+ 04, 3.45E+ 15)	1.20E+ 120, 5.15E+ 04
Cigarettes: Cannabis: Alcohol	840.86 (416.29, 1265.44)	1.09E+ 180 (2.07E+ 89, 5.78E+ 270)	Infinity, 4.14E+ 89
Cigarettes: Cannabis: Analgesics	966.38 (184.69, 1748.06)	8.18E+ 206 (7.64E+ 39, Infinity)	Infinity, 1.52E+ 40
***Time Lagged Models***			
***Full model - 4 Lags - Just Lagging Cannabis***			
Cigarettes: Cannabis: Analgesics	261.1 (19.06, 503.15)	8.26E+ 39 (0.07, 9.56E+ 80)	1.65E+ 40, 1.00
***Full model - 6 Lags - Just Lagging Cannabis***			
Cannabis	8.49 (1.47, 15.5)	50.45 (1.98, 1.28E+ 03)	100.41, 3.39
***Full Model - 1 Temporal Lag***			
Cannabis	7.88 (1.7, 14.06)	48.60 (2.32, 1.016E+ 03)	96.68, 4.07
Cigarettes: Cannabis: Alcohol	182.23 (29.55, 334.9)	1..00E+ 39 (2.45E+ 06, 4.07E+ 71)	1.99E+ 39, 4.91E+ 06
***Full Model - 2 Temporal Lags***			
Cigarettes: Cannabis: Alcohol	658.72 (396.6, 920.84)	3.76E+ 140 (5.65E+ 84, 2.53E+ 196)	7.58E+ 140, 1.13E+ 85
***Full Model - 4 Temporal Lags***			
Cigarettes: Cannabis: Alcohol	472.69 (145.49, 799.88)	9.42E+ 126 (2.81E+ 30, 3.15E+ 223)	1.88E+ 127, 5.62E+ 30
***Space-Time Lagged Models***			
***Full Model - 1 Spatial & 1 Temporal Lag***			
Cannabis: Analgesics	110.36 (37.53, 183.19)	2.51E+ 23 (9.78E+ 07, 6.48E+ 38)	5.03E+ 23, 1.95E+ 08
Cigarettes: Cannabis: Alcohol	1688.83 (336.9, 3040.77)	Infinity (1.033E+ 72, Infinity)	Infinity, 2.07E+ 72
***Full Model - 2 Spatial & 2 Temporal Lags***			
Cigarettes: Cannabis: Alcohol: Analgesics	3954.04 (1565.01, 6343.08)	Infinity (Infinity, Infinity)	Infinity, Infinity
***Cannabinoid Models***			
***Cannabinoids as Main Effects***			
Cigarettes: Δ9THC: Analgesics	391 (181.28, 600.72)	1.62E+ 84 (1.65E+ 39, 1.59E+ 129)	3.24E+ 84, 3.30E+ 39
Δ9THC: Alcohol	116 (51.71, 180.29)	1.11E+ 25 (1.68E+ 11, 7.41E+ 38)	2.23E+ 25, 3.36E+ 11
Cigarettes: Δ9THC: Cannabigerol: Alcohol	4810 (2124.8, 7495.2)	Infinity (Infinity, Infinity)	Infinity, Infinity
Δ9THC: Cannabigerol	109 (41.58, 176.42)	2.45E+ 23 (7.67E+ 08, 7.83E+ 37)	4.90E+ 23, 1.54E+ 09
Δ9THC: Cannabigerol: Alcohol: Analgesics	5640 (1680.8, 9599.2)	Infinity (Infinity, Infinity)	Infinity, Infinity
Cigarettes: Δ9THC	9.01 (0.6, 17.42)	87.15 (1.35, 5.61E+ 03)	173.80, 2.04
***Cannabinoids as Main Effects - 2 Lags***			
Cannabigerol	21.6 (9.29, 33.9)	3.32E+ 04 (89.18, 1.23E+ 07)	6.64E+ 04, 177.84
THC: Alcohol	10.25 (4.12, 16.37)	139.58 (7.34, 2.65E+ 03)	278.66, 14.15
Cannabigerol: Alcohol: Analgesics	1176.24 (308.66, 2043.82)	1.66E+ 246 (9.51E+ 64, Infinity)	Infinity, 1.91E+ 65
***Cannabinoids as Main Effects - 4 Lags***			
Cigarettes: THC	2.87 (1.47, 4.27)	4.06 (2.06, 8.04)	7.58, 3.52
Cigarettes: Cannabigerol: Alcohol	668.38 (191.09, 1145.67)	5.01E+ 141 (5.21E+ 40, 4.82E+ 242)	1.00E+ 142, 1.04E+ 41
***Cannabinoids as Main Effects - 6 Lags***			
Cigarettes: THC	28.16 (18.61, 37.71)	7.61E+ 05 (7.76E+ 03, 7.46E+ 07)	1.52E+ 06, 1.55E+ 04
THC: Cannabigerol	46.22 (30.06, 62.38)	4.50E+ 09 (1.92E+ 06, 1.06E+ 13)	9.01E+ 09, 3.84E+ 06
Cigarettes: Cannabigerol: Alcohol	724.22 (143.74, 1304.71)	1.82E+ 151 (1.84E+ 30, 1.80E+ 272)	3.64E+ 151, 3.68E+ 30
***Ethnicity Models***			
***Cancer Incidence as a Function of Racial Cannabis Exposure***			
Afric-Am._THC_Exp: Hispan.Am_THC_Exp	1.74 (1.18, 2.29)	2.22 (1.72, 2.86)	3.86, 2.86
Afric-Am._THC_Exp: Hispan.Am_THC_Exp: Asian-Am._THC_Exp: AIAN-Am._THC_Exp	0.15 (0.09, 0.21)	1.51 (1.18, 1.91)	2.38, 1.66
Asian-Am._THC_Exp: AIAN-Am._THC_Exp	0.89 (0.37, 1.41)	1.06 (1.04, 1.10)	1.34, 1.24

Table 12 lists 56 E-Values related to cannabis or cannabinoids of which 24 are larger than 1000. Of the 33 E-Values originating from geospatial models, 20 are larger than 1000. The table lists six minimum e-Values of infinity, three deriving from mixed effects models and three from geospatial models.

**Fig. 10:**
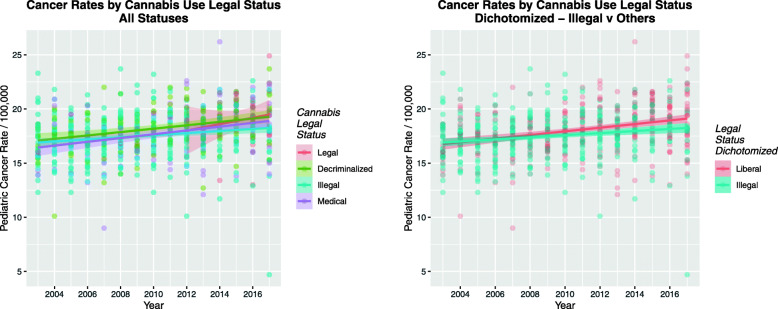
Effect of Cannabis Legal Status on total pediatric cancer incidence rate. **a** Scatterplot of legal statuses over time. **b** Scatterplot of legal status over time dichotomized as illegal status vs. liberal regimes

Given the above compelling data demonstrating a link between rising rates of cannabis exposure and rising TPCIR an obvious extension of this study was whether the increasing use, availability and concentration of cannabis associated with more liberal legal paradigms [57] was associated with elevated TPCIR . One important caveat on such an investigation is that since the data only run to 2017 and many populous states had not yet been affected by the cannabis legalization movement, it may be considered that the data is premature for a full determination of this potential effect. Fig. 10a shows the rate of TPCIR under various legal paradigms. Whilst the few states involved with full cannabis legalization at that time were associated with broad confidence interval bands there is a clear impression in this Figure that the rate under decriminalization appeared to be at a higher levels than others. Fig. 10b dichotomizes the data into liberal paradigms vs. traditional policies of cannabis being considered illegal. Separation of the two regression lines towards the right hand side of the graph gives a clear impression for a significant interaction between time and dichotomized legal status.

These differences are formally assessed in Table 13 by linear regression. Decriminalized and legal status are both confirmed to be significant on their own (upper table segment). In interaction with time decriminalized status is significant (middle table segment). Dichotomized legal status is also found to be significant in interaction with time (lower table segment, β-estimate = 1.87 × 10^− 4^, (2.9 × 10^− 5^, 2.45 × 10^− 4^), *P* = 0.0208). Table 12 lists the minimum E-Values associated with these changes as 1.60 and 1.98 for cannabis decriminalization and full cannabis legalization respectively (at the bottom of the Linear Regression part of Table 12).

**Table 13:** Linear Regressions for Legal Status

Parameter Estimates			Model Parameters			
Parameter	Estimate (C.I.)	***P***-Value	R-Squared	F	dF	***P***-Value
***Cancer by Status***						
***lm(Cancer_Rate ~ Legal_Status)***						
Decriminalized	0.78 (0.37, 1.19)	2.0E-04	0.0268	7.88	3746	3.49E-05
Legal	1.51 (0.68, 2.35)	4.0E-04				
Cancer by Year * Status						
lm(Cancer_Rate ~ Year * Legal_Status)						
Year	0.13 (0.09, 0.16)	4.3E-11	0.0809	17.5	4745	1.01E-13
Year: Decriminalized	0.0003 (0.0001, 0.0005)	4.4E-03				
Cancer by Year * Dichotomized_Status						
lm(Cancer_Rate ~ Year * Dichotomized_Status)						
Year	0.128 (0.09, 0.16)	9.8E-12	0.0778	32.6358	2747	2.58E-14
Year: Liberal	0.0002 (0, 0.0004)	2.1E-02				

## Discussion

### Main results

The main results of this study confirmed that total Pediatric cancer rates have risen significantly nationally across USA and this trend holds for the commonest pediatric malignancies the leukaemias, Non-Hodgkins lymphoma, localized and distant sarcoma and testicular cancer. It was important to note across this period that the use of tobacco, alcohol use disorders, cocaine and analgesic abuse declined as measured in major national surveys whilst cannabis use alone was rising. The level of cannabinoids identified in Federal seizure data also rose for most cannabinoid analytes. TPCIR rose strongly and significantly as a function of cannabinoid exposure, but only weakly and non-significantly in bivariate analysis in relation to cannabis itself. TPCIR was significantly higher in the two highest cannabis use quintiles both overall and across time. Inverse probability weighting was used to equilibrate cannabis exposure across the cohort. Indices of ethnic cannabinoid exposure and seizure cannabinoid concentrations were variously used as instrumental variables to adjust panel models.

Cannabis use was independently associated with TPCIR in additive robust marginal structural, mixed effects, panel and geospatiotemporal models. Cannabis use was independently associated with TPCIR in interactive mixed effects and geospatial models. Cannabis use was linked with TPCIR in various interactions in linear models, robust marginal, mixed effects, panel and geospatial models. Cannabis was independently linked with TPCIR in geospatial models lagged to zero, 1 and 6 years and featured in interactions lagged to 1,2,4 and 6 years. When the cannabinoids THC and cannabigerol were studied they were also linked with TPCIR at high levels of statistical significance at zero, 2, 4 and 6 years of lag.

On sensitivity analysis 49 of 56 minimum e-Values were above 1.25 which is a quoted threshold for likely causal relationships. Similarly 31 of 33 geospatial e-Values were above this threshold. The highest finite minimum e-Value was 4.14 × 10^89^. Six minimum e-Values were infinity.

The recent trend to cannabis liberalization was associated with elevated TPCIR both as a group and as an acceleration of the time-dependent trend in cannabis-liberal states.

Our interpretation of these highly consistent and concordant findings obtained by several methodologies with instrumental variables, controlling for ethnic cannabinoid exposure, utilizing robust regression techniques, inverse probability weighting with high levels of association across both space and time together with very high e-Values is that the relationship of cannabinoid exposure to total pediatric cancer incidence fulfills the criteria of causality and explains the increasing rates of pediatric cancer under cannabis-liberal legislative paradigms, and that this statement is especially true for THC and cannabigerol the two cannabinoids which show the most consistent rises over time.

Hence our study is closely concordant with other published series on the link between pediatric cancer and cannabis use [7–11].

### Statistical comments and causal assignment

It is worth considering briefly the incisive logical power of space-time regression and commenting concisely on the theoretical underpinning of formal causal inferential techniques. To say that two variables are statistically associated carries a certain weight. To say that two variables are closely associated when their distribution is considered across both space and time simultaneously is strongly suggestive of a presumptively causal relationship.

Nineteen spatiotemporal models were presented. In seventeen the spatial error coefficient rho was significant. In eighteen the spatial error autocorrelation coefficient lambda was significant. And spatial errors adjusted in the manner of Kapoor, Kelejian and Prucha consistently had higher precision than those adjusted by the algorithm of Baltagi. The Kapoor, Kelejian and Prucha adjustment accounts for correlation between spatially correlated outcomes in addition to spatially correlated exposures. The presence of clear evidence for spatiotemporal progression of the risk factors (cannabis exposure) together with the many associations shared by the four US regions make spatiotemporal autocorrelation in both the exposure and the outcome a reasonable analytical presumption. Together this is indisputable evidence of effects operating in a spatially distributed manner, and represents in the data analytical environment a reflection of the orchestrated campaign across USA to legalize cannabis which operated in a coordinated manner from the west coast eastwards.

Some comments in relation to casual inference and causal assignment are pertinent. Inverse probability weighting is a method which is well established to correct for inconsistent exposures amongst groups. It is enjoys a strong theoretical and epidemiological evidence base [58]. One of the most serious and common limitations of observational studies is where differential exposure to the risk factor occurs differs across experimental groups. In the typical experimental scenario if the exposure of interest occurs differently between the control and treatment groups then the effect of treatment is necessarily confounded by the non-random risk exposure. The established technique of inverse probability weighting overcomes this major obstacle by having the effect of evening out the exposure of interest across all the groups therefore transforming a merely observational and potentially biased study into a pseudo-randomized trial design where causal inferences can more properly be drawn from group comparisons. The techniques of inverse probability weighting can also be extended to studies where the exposure occurs along a continuum as in the present study. One notes that all of our mixed effects, robust regression and panel models were inverse probability weighted so that they all enjoyed the advantage of this powerful modern innovation. Analysis of such models therefore allows truly causal conclusions to properly be drawn.

Similarly E-Values were recently introduced in a formal way to quantitate extraneous uncontrolled confounding from unmeasured covariates and provides a quantitative magnitude to the level of association required of unknown factors with both the exposure and the outcome to remove the impact of any described association [59]. The E-Value is expressed on the risk ratio scale. It is a classical criticism of multivariable studies that potentially the inclusion of other covariates beyond those which were measured might account for the observed effect. By quantitating the magnitude of the association required with both the exposure of interest and the outcome observed E-Values provide a quantitative measure of the magnitude of the effect which would be required. Very large E-Values necessarily imply that in the absence of some known major confounder uncontrolled confounding becomes exceedingly unlikely and the reported effect becomes more likely to be truly causal in nature. That is large E-Values are associated with truly causal effects [56, 59].

The published literature on E-Values reports that levels in excess of 1.25 are generally taken in the literature as implying causal relationships [56]. By comparison the E-value for the relationship between tobacco smoking and lung cancer is 9.0 [60]. This is considered a very large effect [56, 59]. Hence in our study where 49/53 E-Values were > 1.25, 33 were > 5 and six were infinite discussion of truly causal effects is also entirely appropriate. One notes further that data on some covariates, such as environmental pollution, dietary habits, education, parental age and prematurity rates was not available to the present analytical team. However in view of the very large size of the E-values presently reported it is felt to be highly unlikely that inclusion of further covariates would substantially alter the major conclusions of the present investigation. Naturally we would however be keen to see our studies extended by other groups who have access to more comprehensive datasets defined across space and time.

Our argument for causality relies not just upon the strength of the individual components of the cumulative case but upon their synergistic and syllogistic supporting and reinforcing relationship with each other.

### Pathways and mechanisms

Of pivotal importance in linking associational findings with causal pathways is the issue of biological plausibility and the cellular and molecular pathways which might connect the exposure of interest with the outcome of concern. The subject of the pro-oncogenic activities and potential of cannabis, cannabis smoke and cannabinoids is complex major papers have addressed this issue [14, 26, 28, 32, 34–36, 42, 61–67]. In this paper we will provide a brief and concise overview of what presently seem to be some of the most important pathways which are likely to be implicated. They will be described under nine headings of: gametotoxicity, genotoxicity, epigenotoxicity, mitochondriopathy, immunomodulation, pro-aging, endovascular ischaemia – hypoxia, sympathetically mediated effects on stem cell niches and non-linearity of the dose-response genotoxic effect curve. These domains are not independent but are themselves interdependent and intricately intertwined. Whilst most of the following observations have been experimentally defined the logical sequence has been filled out where this seems reasonable and concordant with the evidence base.

Cannabinoids have been detected in seminal fluid and have been linked with DNA nicking and fragmentation, abnormal sperm nuclear size, gross abnormalities of sperm morphology including sperm fragmentation, disordered DNA packing and re-packing, disorders of protamine synthesis, histone-protamine substitution and major disruption of sperm DNA methylation [15–17, 31, 37, 62, 68, 69]. Cannabinoids have been found in Graafian follicle and oviduct fluid and have been linked with oocyte nuclear blebbing, nuclear bridging, chromosomal fragmentation and large scale oocyte loss after the second meiotic cell division [14, 15, 17]. Cannabis smoke is known to contain all of the carcinogens of tobacco smoke including many tars and carcinogens including aromatic amines, polycyclic hydrocarbons, and tars [70]. Cannabinoid exposure has been linked with nuclear bleb and chromosomal bridge formation, chromosomal mis-segregation at the anaphase separation, micronucleus formation [71], transposon activation and chain and ring chromosome formation [14, 32, 34]. Cannabidiol, Cannabinol and THC have been implicated in in chromosomal translocation formation to the same level seen with cytotoxic drugs [13]. Cannabidiol and cannabidivarin have been shown to cause double stranded DNA breaks, micronucleus formation and nuclear buds and bridges in human cells which is worse under oxidative stress [67]. Cannabinoid-induced micronucleus formation is very important as it has been identified as a major engine of catastrophic damage to the genetic material and one-step chromothripsis, chromoanagensis and oncogenic transformation [61, 72, 73]. Cannabinoid exposure has been linked with large scale perturbation of DNA methylation, gross defects in histone synthesis – which necessarily leave DNA more open and available for transcription which is a pro-oncogenic state – altered histone signalling, and an inhibition of ATP supply to genetic and epigenetic processes – most of which are energy dependent – and an inhibition of epigenetic substrate supply [31, 33, 35, 37, 62, 74]. Together these changes may be expected to advance the “epigenetic clock” which is believed to be one of the key determinants of cellular aging [75, 76]. The profound implications of major epigenetic reprogramming were highlighted by a recent paper noting that despite the short half life of immune cells in the circulation – just a few days - the cellular basis for long lasting immunity is actually epigenetic changes in long lived myeloid precursor cells which record metabolic and immune activation responses in the coordinated patterns of their enhancers, promoters, long non-coding RNA’s, DNA methylation and histone codes which determine chromatin conformation and the assembly of topologically transcriptionally active domains which functionally facilitate secondary responses to infection and vaccines [77, 78].

The outer mitochondrial membrane not only possess CB1R’s, but indeed the whole of the cannabinoid signalling transduction machinery found in the plasmalemma also resides in the inner and outer mitochondrial membrane and within the intermembrane space so that cannabinoids are an important direct modulator of metabolic state [79–83]. Several adverse mitochondrial processes are well described including a reduction in the transmembrane potential across the inner mitochondrial membrane, a reduced synthesis of key oxidative phosphorylation substrates including the F1-ATPase, increased electron shunting via uncoupling protein 2 activation, gross mitochondrial damage and swelling and impairment of mitonuclear cross-talk and mitonuclear genomic coordination [17, 84–88].

There is a rich literature describing both the pro- and anti- inflammatory actions of cannabinoids. In this context the proinflammatory CB1R-mediated activities seem to be especially important [89] as chronic inflammation is a well established cause of cancers in many tissue beds and occurs by many mechanisms. One pathway of particular interest is that cytoplasmic inflammation stimulates the transposons or “jumping genes” of the genome, to start “jumping” mobile segments and creating genomic havoc. Micronucleus disruption releases double stranded DNA into the cytoplasm where it potently stimulates the cytoplasmic GMP-AMP – STimulator of INterferon Gamma (cGAS-STING) pathway which further intracytoplasmically stimulates inflammation via interferon-γ and innate immune signalling and destabilizes the genome [90–92]. The immunosuppressive activities of cannabinoids may depress the immune response to the developing field change and nascent tumours. This cycle could potentially explain the many case reports of cancers occurring in adults at a younger age than usual and with increased aggressiveness in heavily cannabis exposed patients [93–96].

Cannabis exposure has been found to accelerate organismal cardiovascular aging clinically [97]. Cannabinoids are known to inhibit stem cell division [34, 98]. This combination of impaired stem cell activity, reduction of mitochondrial energy generation and a pro-inflammatory milieu are all hallmarks of cellular ageing and the senescence-associated secretory phenotype [99–101] of growth factors and cytokines which is presumably stimulated and a key hallmark of aging. Aging of course is the leading risk factor for most adult tumours. In the light of the foregoing cellular changes it would seem that the quality of cannabinoid-exposed gametes may be broadly seen as defective and they may thus be said in general terms to likely be “aged” in metabolic, epigenetic and genetic terms. Cannabinoids are known to have important effects on the microvasculature and can induce tissue ischaemia [102–105] which is an important determinant of the hypoxic microenvironment which stimulates genomic instability and oncogenesis and promotes nascent and mature tumour growth. Cannabis addiction is known to feature periods of cannabinoid withdrawal marked by agitation and manifest sympathetic hyperstimulation [106]. Sympathetic stimulation has been shown to have direct adverse activities on the stem cell niche of the hair follicle [107] and likely acts similarly in other stem cell niches.

Arguably the most concerning feature of this literature is the apparent threshold effect beyond which genotoxic and mitochondriopathic changes emerge relatively abruptly. This implies that the exponential dose-effect curve seen in many genotoxic assays for cannabinoids [35, 63, 108, 109] can appear to be functionally an abrupt discontinuity in the dose-response curve at the epidemiological level. At the community level this implies that a doubling of daily cannabis use, as has been documented in USA in recent years [110], might reasonably be linked with a disproportionate response in genotoxic downstream sequaelae such as congenital anomalies including transgenerationally transmissible carcinogenesis.

From this brief overview it is apparent that a plethora of cellular oncogenic mechanisms exist linking exposure to cannabis smoke, cannabis and cannabinoids to the processes of carcinogenesis.

In 1965 Hill described nine criteria as being required of any association in order to assign causality to the relationship. Strength of association, consistency amongst studies, specificity, temporal sequence, coherence with known data, biological plausibility a biological response or dose-response curve, analogy with similar situations elsewhere and experimental confirmation were key features [111]. It will be noted that the above analysis, including the published literature and the cited experimentally demonstrated mechanistic links, fulfill all of these criteria for the relationship between cannabis exposure and TPCIR .

### Generalizability

Our data are population level data derived from publicly available datasets from one of the world’s most technologically advanced nations. The underlying population is also substantial. Given that our findings are robust to various different methods, fulfill criteria for causality and are consistent with the majority of the published work in the area we believe that our findings are robust and widely generalizable. However as it is clear that cannabis use is in a state of flux worldwide at the present with rises in the prevalence of use, intensity of use, and concentration of product we feel that it is important that on-going studies be conducted in this area to monitor the situation at higher levels of geospatial resolution.

### Future directions

Further extensions of this work might include detailed dissection of the molecular and cellular level of the pathways mentioned particularly relating to mitochondrial cannabinoid signalling, mitochondrial electron leaks and shunts, free oxyradical flux, perturbation of mitonuclear cross-talk, cannabinoid induced disruption of metabolic supply of epigenetic substrates, cannabinoid-related disruption of histone synthesis and signalling and the histone code generally, cannabinoid epigenotoxicity generally and heritable and transgenerational epigenotoxicity specifically, proinflammatory cannabinoid actions, microvascular-disrupting and hypoxia-inducing actions, chromosomal mis-segregation and anaphase disruption and the interaction of cannabinoids with the cGAS-STING cytoplasmic signalling pathway. Research into cannabinoid interactions with the germ cells, oocytes and sperm, is clearly of primary and foundational importance to these concerns and should be up-prioritized on research agendas. Analytically higher resolution space-time modelling based on more detailed datasets from CDC and SAMHSA is an obvious task for the near future. The incorporation of instrumental variables and inverse probability weights into the space-time and spatiotemporally lagged models of plm, splm and similar software would allow all the questions of interest to be addressed in a single modelling framework without the need for multiple model types as was necessitated in the present report and would likely only require minimal resources to enable the required programming code to be written for this very impressive, sophisticated and highly flexible software to be further optimized.

### Strengths and limitations

Our study has several strengths including using data from a very populous nation, the use of publicly available datasets, the use of different statistical techniques, the application of inverse probability weighting and e-Values, two mechanisms well established in the causal epidemiological literature, the use of geospatiotemporal regression techniques with complex random error structures, the use of models lagged both spatially and temporally, the use of a variety of covariates, consideration of substance-exposure indices which is often absent from many studies, the use of various instrumental variables, the availability of a relatively lengthy panel data series for 15 years, and correction for ethnic cannabis exposure as a major underlying confounding factor. The absence of geospatial techniques from much of cancer epidemiology appears to be a major knowledge gap which the present study begins to redress. It may also be argued that for research enterprises to consume significant public resources but never be able to provide actual causal advice to their host community at once stretches public credulity and tests their patience, particularly when well established methodologies are available which can be used to fill this major knowledge gap. The deliberate application of the techniques of formal causal inference in this study thus comprises a major strength. The study’s major limitation relates to the unavailability of individual patient-level data which is a common limitation amongst epidemiological studies. Due to the complexity of the present analysis we have not considered further subgroup analyses, either of individual tumours, or by fascinating sex or ethnic incidence differences. All of this remains to be done at higher geospatial resolution by subsequent investigators. Data relating to other covariates such as levels of environmental pollutants, parental age at childrens’ birth, dietary changes and prematurity rates was not available to the present study. These areas remain to be addressed by subsequent researchers. On a methodological note one notes that since cannabis use an effect both educational attainment and occupational achievement one must be careful to avoid over-controlling or the use of “collider variables” in such future regression studies [60, 112].

## Conclusion

In summary our study confirms previous reports in the literature linking cannabis exposure with pediatric and testicular cancer [7–11, 18–22] and answers both our opening hypotheses affirmatively. We extend and amplify earlier reports in many ways including with the use of national cancer census data and widely cited nationally representative drug use surveys, the application of geospatial techniques and the formal techniques of causal inference to the data series and various technical refinements including the use of several sets of instrumental variables and various forms of inverse probability-weighted and spatially weighted regression matrices and robust, panel and linear multivariable techniques. After including socioeconomic, ethnic and drug use variables we find robust associations across space and time for cannabis use and TPCIR and that cannabis, and particularly the cannabinoids THC and cannabigerol, are independently and interactively associated with TPCIR both in de novo space-time grids and in spatially and temporally lagged models. Moreover very high e-Values clearly indicate that the relationship cannot be explained away by unmeasured, unknown or hypothetical confounding variables. This analysis is consistent with five previously reported series comprising the majority of the published literature in the field [7–11], dozens of potential experimentally described mechanistic pathways and fulfill the paradigmatic Hill criteria of causality [111]. Findings are also consistent with reports of elevated rates of congenital anomalies following prenatal cannabis exposure [25–28, 42, 43] and thus are broadly concordant conceptually with wide ranging and far reaching heritable cannabinoid-related genotoxicity. Our analysis also begins to provide insights into the previously mysterious major differences in cancer incidence between various ethnicities by indicating that varying ethnic exposures to cannabinoids are of particular concern. It is important that this thread be further explored in the future. Such formal demonstration of strong evidence of a presumptively genotoxic cannabis-cancer causal link is highly relevant for the ongoing and currently controversial story of the relationship of cannabis use with malignant tumourigenesis in adults. Strong evidence of a robust causal relationship of cannabis exposure to pediatric and thus transgenerational inheritable genotoxicity carries far reaching implications for the ongoing public debate relating to the most appropriate forms of regulation of cannabis and cannabinoids. Moreover the present analysis powerfully informs the broader discussion regarding cannabis-related genotoxicity as it relates to adult tumourigenesis and many congenital anomalies encountered at birth [25–28, 42, 61, 62].

## Supplementary Information

**Additional file 1: Supplementary Figure S1**. Selected and Major Paediatric Cancer Rates Over Time.

**Additional file 2: Supplementary Table S1**. Cannabis Quintile Data

## Data Availability

All data generated or analysed during this study are included in this published article and its supplementary information files. No permissions are required to access the data which was used and collated in this study, e.g. NSDUH study. Data including shapefiles and R programming script is made publicly available on the Mendeley Data Archive at this URL: URL: 10.17632/cnwv9hdspd.1.

## Abbreviations

ACC: All Childhood Cancer
AIAN: American Indian / Alaskan Native
ALL: Acute Lymphoid Leukaemia
CB1R: Cannabinoid Receptor type 1
CBD: Cannabidiol
CBG: Cannabigerol
CDC: Centres for Disease Control
cGAS: Cyclic Guanosine-Adenosine Mononucleotide synthase
cGAS-STING: Cytoplasmic GMP-AMP – STimulator of INterferon Gamma
CRAN: Comprehensive R Archive Network
DNA: Deoxyribonucleic acid
E-Value: Expected Value
F1-ATPase: Mitochondrial F1-ATP Synthase
NHPI: Native Hawaiian / Pacific Islander
NSDUH: National Survey of Drug Use and Health
PCE: Prenatal Cannabis Exposure
RDAS: Restricted Use Data Analysis System
SAMHDA: Substance Abuse and Mental Health Data Archive
SAMHSA: Substance Abuse and Mental Health Services Administration
SEER: Surveillance Epidemiology and End Results
STING: STimulator of INterferon Gamma
THC: Δ9-Tetrahydrocannabinol
TPCIR: Total Pediatric Cancer Incidence Rate
