# Supplementary figures and images for: A geospatiotemporal and causal inference epidemiological exploration of substance and cannabinoid exposure as drivers of rising US pediatric cancer rates
Source: BMC Cancer. 2021 Feb 25;21:197. doi: 10.1186/s12885-021-07924-3 (PMC7908679; doi:10.1186/s12885-021-07924-3)

# Selected and Major Paediatric Cancer Rates Over Time

## 21 US Cancer Registries

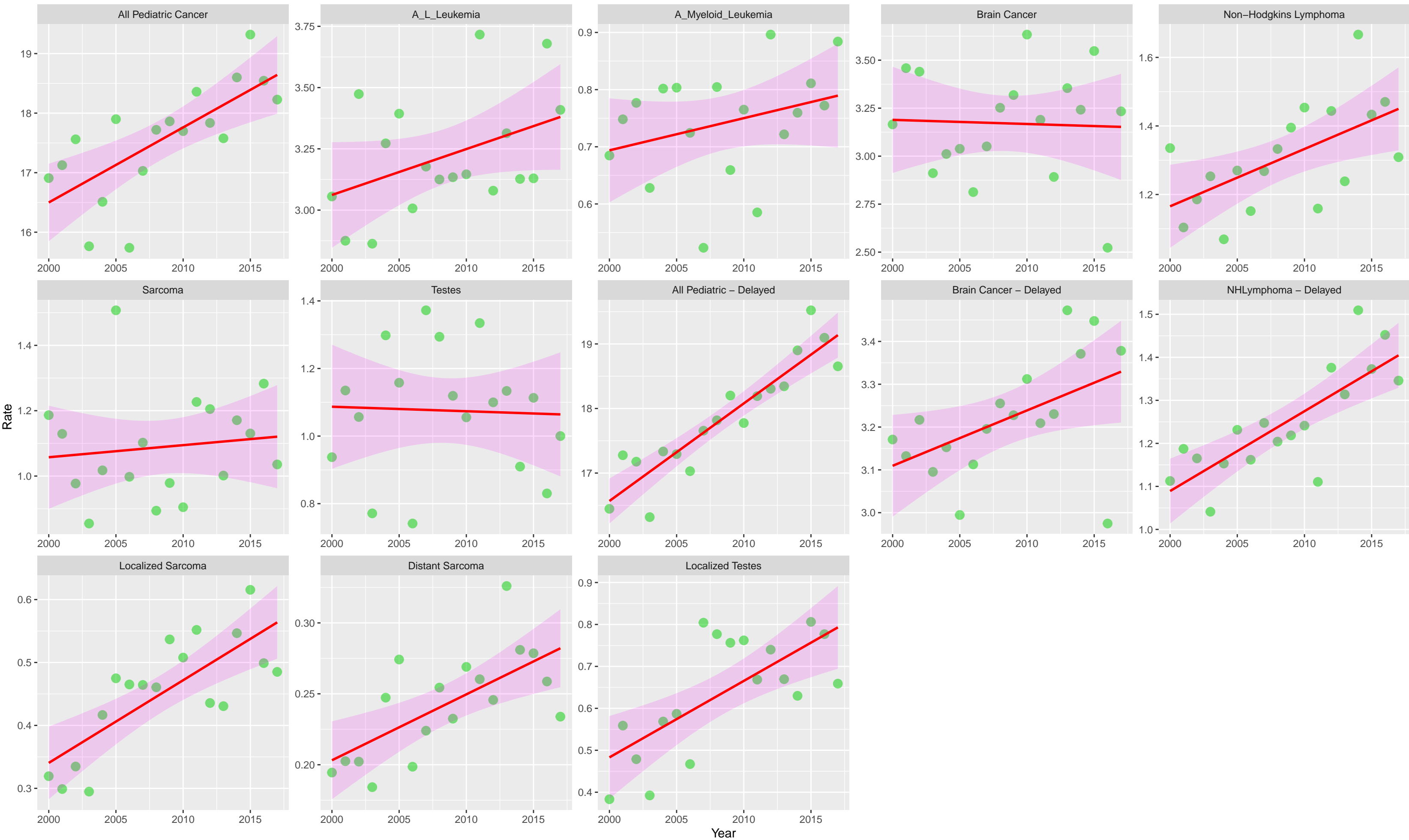

Supplement: Supplementary file 1 — Additional file 1: Supplementary Figure S1. Selected and Major Paediatric Cancer Rates Over Time. [file 12885_2021_7924_MOESM1_ESM.pdf]
