# Supplementary material for: A geospatiotemporal and causal inference epidemiological exploration of substance and cannabinoid exposure as drivers of rising US pediatric cancer rates
Source: BMC Cancer. 2021 Feb 25;21:197. doi: 10.1186/s12885-021-07924-3 (PMC7908679; doi:10.1186/s12885-021-07924-3)
Supplement: Supplementary file 2 — Additional file 2: Supplementary Table S1. Cannabis Quintile Data [file 12885_2021_7924_MOESM2_ESM.docx]

**Supplementary Table 1.: Cannabis Quintile Data**

| **Quintile** | **Cannabis Exposure** | **Cancer Rates** |
| --- | --- | --- |
|  |  |  |
| **Quintiles** |  |  |
| Quintile 1 | 0.1101 (0.0038) | 17.2941 (0.1913) |
| Quintile 2 | 0.1349 (0.0042) | 17.4933 (0.1763) |
| Quintile 3 | 0.1552 (0.0044) | 17.4381 (0.1806) |
| Quintile 4 | 0.1731 (0.005) | 18.0087 (0.1581) |
| Quintile 5 | 0.2304 (0.0062) | 18.6060 (0.1767) |
|  |  |  |
| **Dichotomized Quintiles** |  |  |
| Lower Quintiles | 0.1331 (0.0025) | 17.4076 (0.1055) |
| Upper Quintiles | 0.2018 (0.0043) | 18.3073 (0.1196) |
